# Short-term association of CO and NO_2_ with hospital visits for glomerulonephritis in Hefei, China: a time series study

**DOI:** 10.3389/fpubh.2023.1239378

**Published:** 2023-08-21

**Authors:** Haifeng Chen, Qiong Duan, Huahui Zhu, Shuai Wan, Xinyi Zhao, Dongqing Ye, Xinyu Fang

**Affiliations:** ^1^Department of Epidemiology and Biostatistics, School of Public Health, Anhui Medical University, Hefei, Anhui, China; ^2^Inflammation and Immune Mediated Diseases Laboratory of Anhui Province, Hefei, Anhui, China; ^3^Department of Health Management Center, The First Affiliated Hospital of Anhui Medical University, Hefei, Anhui, China

**Keywords:** air pollution, glomerulonephritis, carbon monoxide, nitrogen dioxide, distributed lag nonlinear model, time-series study air pollution, time-series study

## Abstract

**Objective:**

Recent studies suggest air pollution as an underlying factor to kidney disease. However, there is still limited knowledge about the short-term correlation between glomerulonephritis (GN) and air pollution. Thus, we aim to fill this research gap by investigating the short-term correlation between GN clinical visits and air pollution exposure.

**Methods:**

Between 2015 and 2019, daily GN visit data from two grade A tertiary hospitals in Hefei City were collected, along with corresponding air pollution and meteorological data. A generalized linear model integrated with a distributed lag nonlinear model was employed to analyze the relationship between GN visits and air pollutants. Moreover, we incorporated a dual pollutant model to account for the combined effects of multiple pollutants. Furthermore, subgroup analyses were performed to identify vulnerable populations based on gender, age, and season.

**Results:**

The association between 23,475 GN visits and air pollutants was assessed, and significant positive associations were found between CO and NO_2_ exposure and GN visit risk. The single-day lagged effect model for CO showed increased risks for GN visits from lag0 (RR: 1.129, 95% CI: 1.031–1.236) to lag2 (RR: 1.034, 95% CI: 1.011–1.022), with the highest risk at lag0. In contrast, NO_2_ displayed a more persistent impact (lag1–lag4) on GN visit risk, peaking at lag2 (RR: 1.017, 95% CI: 1.011–1.022). Within the dual-pollutant model, the significance persisted for both CO and NO_2_ after adjusting for each other. Subgroup analyses showed that the cumulative harm of CO was greater in the cold-season and older adult groups. Meanwhile, the female group was more vulnerable to the harmful effects of cumulative exposure to NO_2_.

**Conclusion:**

Our study indicated that CO and NO_2_ exposure can raise the risk of GN visits, and female and older adult populations exhibited greater susceptibility.

## 1. Introduction

Glomerulonephritis (GN) is a heterogeneous collection of diseases identified by inflammatory damage within the renal small vessels and glomeruli (1). Ranking as the third and second leading causes of chronic kidney disease (CKD) (2) and end-stage renal disease (ESRD) (3) worldwide, GN has witnessed a considerable escalation in disease burden, with 6.9 million disability-adjusted life years (DALYs) due to CKD brought about GN in 2019, representing a 66% increase compared to 1990 (2). Despite progress in the nosogenesis of GN, the etiology of GN remains elusive. In recent years, environmental factors, notably air pollution, have been increasingly recognized as potential factors to the occurrence and progression of kidney diseases, including GN (4–8).

In a groundbreaking nanomedicine study (9), researchers have shown initial evidence of inhaled aerosol particles’ potential to interact with renal tissue. The investigation revealed that gold nanoparticles with a diameter of ≤4 nm, when inhaled, can penetrate alveolar tissue, subsequently entering the bloodstream and being detected in participants’ urine within three months. Supporting these findings, *in vivo* experiments (10–12) have demonstrated that sustained exposure to fine particulate matter of 2.5 μm or less in diameter (PM_2.5_) may elicit renal inflammatory responses, structural damage, and oxidative stress in the kidneys of rodent models.

Despite these findings, only a few researchers have explored the correlation between atmospheric contaminant exposure and some clinical subtypes of GN. These studies (5–8) have provided some indications that exposure to elevated concentrations of PM_2.5_ or acidic gases increases the risk of developing IgA nephropathy (only for PM_2.5_) and nephrotic syndrome. However, there is still limited knowledge regarding the short-term link between GN and air pollution. Moreover, the combined effects of multiple pollutants and the differences in risk among populations with different demographic characteristics are still unclear.

To fill this research gap and elucidate the potential correlation between exposure to atmospheric contaminants and GN, we inquired into the short-term relationship between GN visits and air pollution exposure based on data from nephrology clinics at two grade a class 3 hospitals in Hefei, China, from 2015 to 2019. In addition, we further identified risk patterns across age and gender to explain the latent impact of discrepancies in susceptibility between subpopulations.

## 2. Materials and methods

### 2.1. Study area

The study was conducted in Hefei City, encompassing four Districts. As the capital of Anhui Province, Hefei spans 11,445 km^2^ and houses approximately 8.19 million inhabitants (13). Situated within the mid-latitude zone (31°N, 117°E), Hefei experiences a humid subtropical monsoon climate typical of the region. As part of the “Yangtze River Delta” city cluster, Hefei is located in the coastal hinterland and serves as a vital industrial economic center. Consequently, the city grapples with significant air pollution issues, resulting in substantial public health concerns (14).

### 2.2. Study population

We amassed daily visit data for GN patients between 2015 and 2019 from the electronic medical record information systems of the First Affiliated Hospital of Anhui Medical University and the First Affiliated USTC. The variables compiled for the GN data encompassed age, gender, outpatient date, and residential address. The diagnostic criteria for GN are as follows: (1) Persistent proteinuria and/or hematuria over an extended period; (2) A history of prolonged hypertension, mild renal impairment, or/and edema; (3) Gradual, relentless progression of renal impairment, culminating in end-stage renal failure in later stages; (4) Symmetrical reduction in kidney size; and (5) Exclusion of secondary chronic nephritis syndrome, which would point toward a primary diagnosis (3, 15). The inclusion criteria for GN patients in this investigation were: (1) adherence to the diagnostic criteria for GN; (2) current address in Hefei; and (3) possession of hospital records. Furthermore, we applied exclusion criteria: (1) concurrent visits or a second visit to both hospitals within a brief period; (2) unclear diagnosis; (3) non-local residents; and (4) patients devoid of demographic information (e.g., age and gender). The data variables utilized in this study were anonymized, negating the need for ethical restrictions due to the absence of risk research requirements.

### 2.3. Air pollution and meteorological data

Air pollution data were procured from the average values of 10 ambient air pollution monitoring stations at the Hefei Environmental Monitoring Center, which included 24-h average levels of particulate matter of 10 μm or less in diameter (PM_10_), PM_2.5_, carbon monoxide (CO), nitrogen dioxide (NO_2_), sulfur dioxide (SO_2_), and daily maximum 8-h average ozone (O_3_-8h) concentration. Concurrently, meteorological data were acquired from the China meteorological data network,^1^ comprising daily average temperature (Temp), air humidity (RH), sunshine duration (SSD), and rainfall (RF).

### 2.4. Statistical analysis

By aggregating daily data, we employed a time-series study to calculate the risk of GN visits associated with short-term exposure to atmospheric contaminants from 2015 to 2019. As GN visits represent a low probability event within the population and exhibit overdispersion (Supplementary Table S1), a typical time series generalized linear model (GLM) with a quasi-Poisson connection was integrated with the distributed lag nonlinear model (DLNM) to determine the relationship between atmospheric contaminants and GN visits (16). The maximum delay on day 7 was utilized to capture the lagged impact of atmospheric contaminants (16).

To circumvent multicollinearity issues and account for the non-normal distribution (Supplementary Table S2) of variables, we performed Spearman correlation analysis on all variables, excluding those with correlation coefficients greater than 0.7 (17). The final model is presented below:


(1) $$\begin{array}{c} \begin{array}{l} \log\left(E\left[Y_{t}\right]\right)=\alpha +\beta cb\left(X_{0}\right)+ns\left(time,dfs\right)+\\ ns\left(meteorologicalfactors,dfs\right) \end{array}\\ \begin{array}{l} +ns\left(air\;pollutions,dfs\right)+as.factor\left(DOW\right)+\\ as.factor\left(holiday\right) \end{array} \end{array}$$

Here, *E*[*Y*_t_] represents the anticipated count of patients with GN on day *t*; cb(X_0_) denotes the cross-basis function of the explanatory variable, which employs the “lin” function and “poly” function (degree = 3) to define the matrices of exposure and lag (0–7), respectively; α signifies the intercept; β stands for the cross-basis function’s coefficient; ns represents the natural cubic spline (ns) function; time refers to the long-term time trend effect; dfs is the degrees of freedom, which is chosen based on the minimum value of the Akaike information criterion (AIC) applicable to the quasi-Poisson model (16). To control potential confounders, we introduced meteorological factors (Temp, RH, SSD, and RF) and other atmospheric contaminants (when X_0_ is CO: NO_2_, SO_2_, O_3_-8h, and PM_10_) with ns function. Furthermore, we incorporated holiday parameters and DOW into the model as categorical variables to control the holiday and day-of-the-week effects.

We incorporated air pollutants individually upon constructing a working model (the core model) containing all control variables and verifying their suitability. Subsequently, we calculated the lag effect of atmospheric contaminants on GN visits from the current day (lag0) to 7 days prior (lag7). Previous research (18) has indicated that single-day lag models may neglect the cumulative effect. Consequently, we integrated the cumulative lag effect analysis into the model. Relative risk (RR) values and 95% confidence interval (CI) of air pollutant concentration per 10 μg/m^3^ increment (1 mg/m^3^ CO) were employed to calculate the short-term correlation between atmospheric contaminants and GN visits. Lastly, we used P50 as the reference base to calculate the association between the single-day effect and the cumulative lag effect of increasing air pollutant concentration on GN visits.

While the single pollutant model can predict the association between one atmospheric contaminant and GN visits, air pollutants often coexist and interact, resulting in a comprehensive impact on human health (19). To assess this potential combined effect and the robustness of the impact caused by pollutants, we established a dual pollutant model to study the intermingling effects of pollutants. The formulation of the dual pollutant model is as under:


(2) $$\begin{array}{c} \begin{array}{l} \log\left(E\left[Y_{t}\right]\right)=\alpha +\beta_{1}cb\left(X_{a}\right)+\beta_{2}cb\left(X_{b}\right)+ns\left(time,dfs\right)\\ +ns\left(air\;pollutions,dfs\right) \end{array}\\ \begin{array}{l} +ns\left(meteorologicalfactors,dfs\right)+as.factor\left(DOW\right)\\ +as.factor\left(holiday\right) \end{array} \end{array}$$

Here, *cb*(*X*_a_) and *cb*(*X*_b_) are the cross-basis functions of the included dual pollutants, and β is the coefficient. All other parameters remain the same as in Model (1).

Additionally, to identify vulnerable populations and seasons, we further stratified the population by gender (male and female), season (cold season: November–April in the next year; warm season: May–October), and age (<65 and ≥ 65 years) to conduct subgroup analyses (20). Subsequently, the Wilcoxon signed-rank test was employed to confirm dissimilarities among the aforementioned subgroups.

### 2.5. Sensitivity analyses

To evaluate the reliability of the results, we carried out sensitivity assessments. First, we varied the dfs (3–8 dfs) (21) for the *time*, *air pollutions*, and *meteorological factors*. Second, we adjusted the maximum delay of air pollutants to intervals of 14 and 21 days to examine the harvesting effect (22, 23). Lastly, we set the reference values of all pollutants to 0, plotted the overall exposure-response curves, and calculated the relative risk (RR) values for the corresponding pollutants.

All statistical computations were performed with the aid of R software (version 4.2.3). Statistical significance of the effect was acknowledged when *p* < 0.05 (two-sided).

## 3. Results

### 3.1. Descriptive analysis

**Figure 1:**
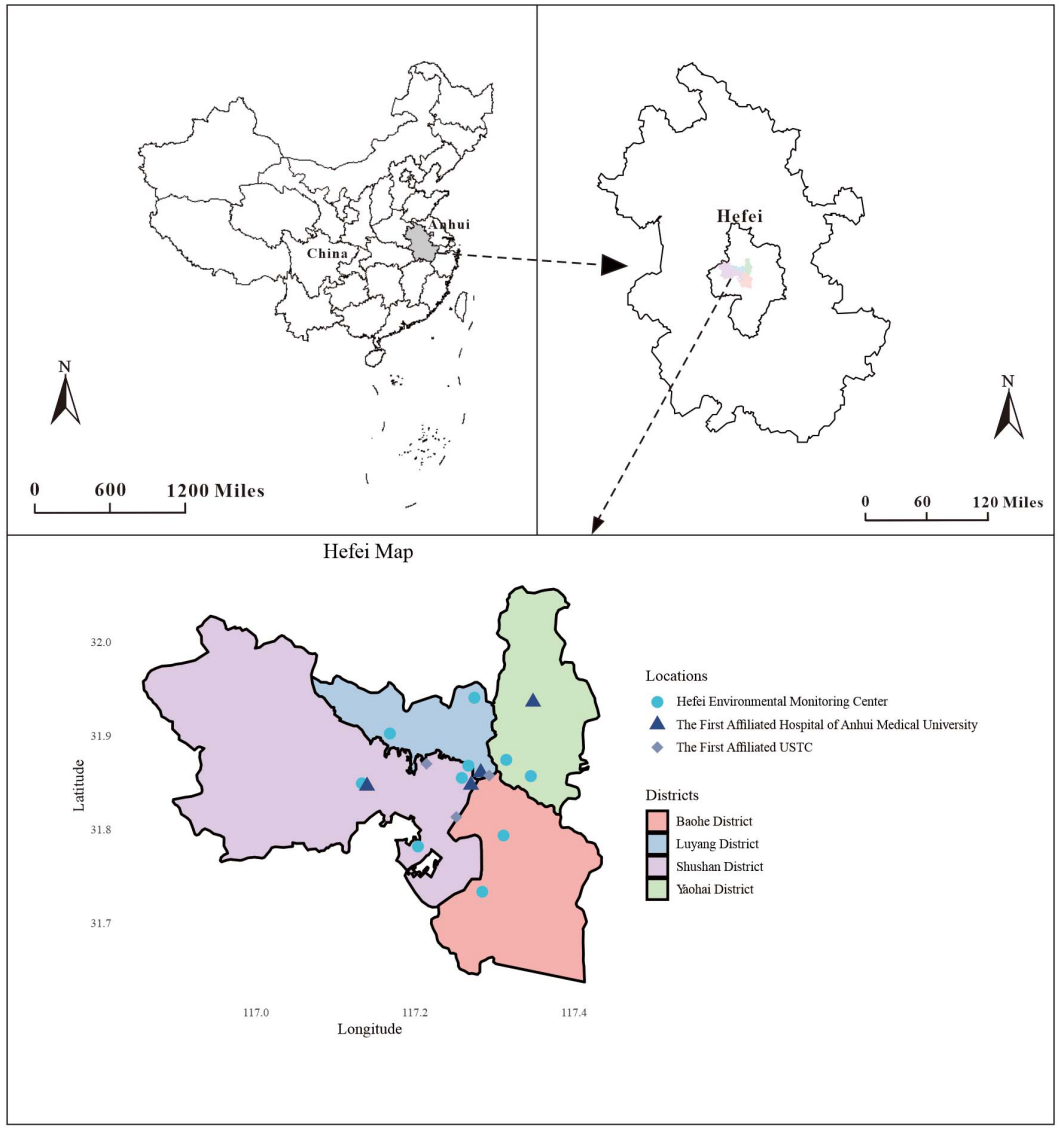
Geographical distribution of environmental monitoring stations and the two hospitals in Hefei.

Between 2015 and 2019, the electronic medical record information system registered 23,475 GN visits at the two hospitals, comprising 47.23% males (11,088), 10.02% older adults aged ≥65 (2,352), and 49.37% occurring during the cold season (11,589). Throughout the research duration, the daily mean levels (with standard deviation) of CO, NO_2_, PM_10_, PM_2.5_, SO_2_, and O_3_-8h were 0.84 (0.28) mg/m^3^, 40.31 (17.76) μg/m^3^, 76.17 (38.90) μg/m^3^, 52.29 (33.41) μg/m^3^, 10.55 (6.08) μg/m^3^, and 86.32 (44.01) μg/m^3^, respectively. Additionally, Temp, RH, SSD, and RF (with standard deviation) were found to be 16.85 (9.21)°C, 76.51 (11.99)%, 4.92 (4.02) h, and 3.18 (8.88) mm, respectively (Table 1). To better illustrate the geographical context of our study, we have included a map showcasing the locations of these two hospitals and 10 ambient air pollution monitoring stations within the city (Figure 1). Besides, the time series distribution of the data over 5 years is illustrated in Supplementary Figure S1. The Spearman correlation analysis revealed a strong correlation between PM_2.5_ with PM_10_ (*r*_s_ = 0.806, *p* < 0.001) and CO (*r*_s_ = 0.812, *p* < 0.001), prompting its exclusion from the model (Supplementary Figure S2).

**Table 1:** Descriptive statistics of the daily outpatient visits for GN, air pollutant concentrations, and meteorological parameters in Hefei City, 2015–2019.

Variables	*n* (%)	Mean ± *sd*	Min	*P*_25_	*P*_50_	*P*_75_	Max
Visits							
Total	23,475 (100.00)	12.86 ± 9.45	0.00	5.00	12.00	19.00	57.00
Gender							
Male	11,088 (47.23)	6.07 ± 4.86	0.00	2.00	5.00	9.00	31.00
Female	12,387 (52.77)	6.78 ± 5.35	0.00	2.00	6.00	10.00	30.00
Age (years)							
<65	21,123 (89.98)	11.57 ± 8.44	0.00	4.00	11.00	17.00	47.00
≥65	2,352 (10.02)	1.29 ± 1.66	0.00	0.00	1.00	2.00	12.00
Season							
Cold	11,589 (49.37)	12.79 ± 9.56	0.00	4.00	12.00	19.00	50.00
Warm	11,886 (50.63)	12.92 ± 9.33	0.00	5.00	12.00	18.00	57.00
Air pollutant concentration							
CO (mg/m^3^)		0.84 ± 0.28	0.30	0.60	0.80	1.00	2.60
NO_2_ (μg/m^3^)		40.31 ± 17.76	9.00	27.00	36.00	51.00	125.00
SO_2_ (μg/m^3^)		10.55 ± 6.08	2.00	6.00	9.00	13.00	51.00
PM_10_ (μg/m^3^)		76.17 ± 38.90	8.00	48.00	71.00	97.00	308.00
PM_2.5_ (μg/m^3^)		52.29 ± 33.41	6.00	29.00	44.00	66.00	237.00
O_3_-8h (μg/m^3^)		86.32 ± 44.01	4.00	52.00	79.00	115.00	241.00
Meteorological conditions							
Temp (°C)		16.85 ± 9.21	−6.20	8.60	17.60	24.50	34.80
RH (%)		76.51 ± 11.99	39.20	68.70	77.00	85.70	98.30
SSD (h)		4.92 ± 4.02	0.00	0.40	5.10	8.50	12.90
RH (mm)		3.18 ± 8.88	0.00	0.00	0.00	1.60	134.00

### 3.2. Overall effects

The overall exposure-response relationship between atmospheric contaminants (CO, NO_2_, PM_10_, SO_2_, and O_3_-8h) and GN visits is predicted by the DLNM model (Supplementary Figure S3). The curves demonstrated a positive correlation between CO and NO_2_ exposure and an escalated risk of GN visits when the maximum lag was 7 days (Supplementary Figures S3A,B). No significant overall exposure-response associations were observed between SO_2_, PM_10_, and O_3_-8h and GN visits, as their 95% CIs included the null value (RR = 1.000) throughout the entire range (Supplementary Figures S3C–E). Consequently, the model included PM_10_, SO_2_, and O_3_-8h as covariates for subsequent analyses to account for potential confounding effects.

**Figure 2:**
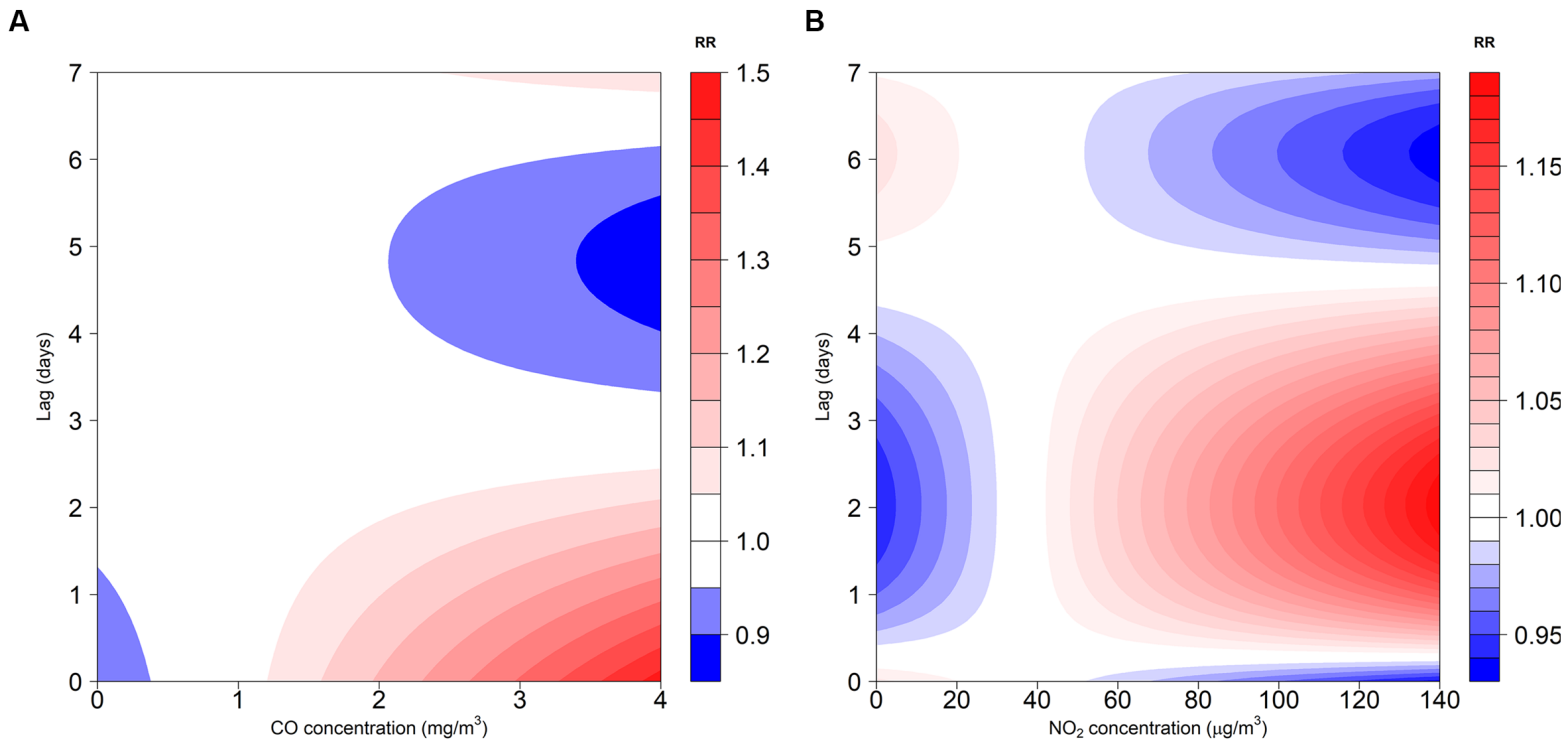
Contour plot for the association between air pollutants (CO and NO_2_) and GN visits in Hefei, China, 2015–2019. **(A)** Contour plot of CO; **(B)** Contour plot of NO_2_. CO, carbon monoxide; NO_2_, nitrogen dioxide; and RR, relative risk. The P_50_ was used as the reference concentration. The P_50_ concentration of CO is 0.8 mg/m^3^ and the P_50_ concentration of NO_2_ is 36 μg/m^3^.

**Figure 3:**
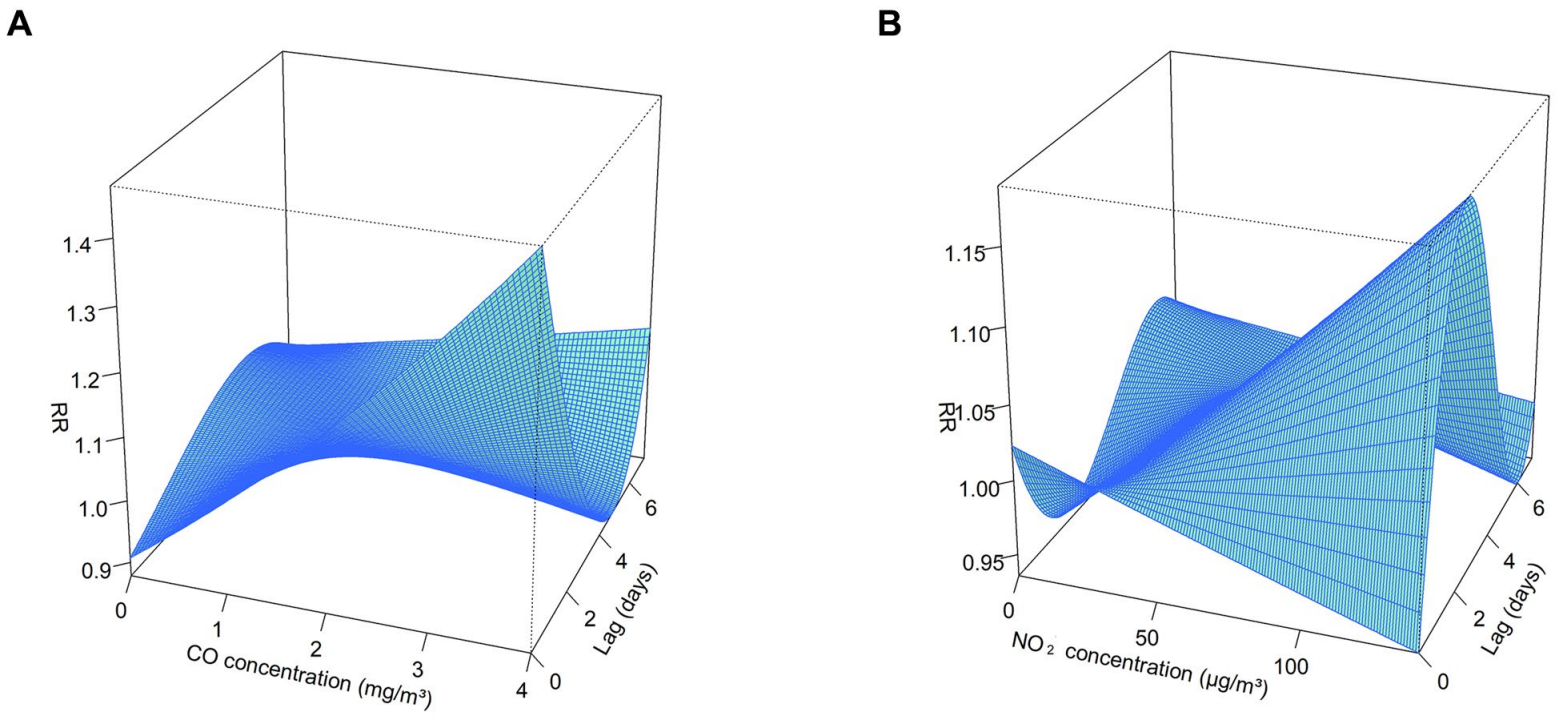
3D plot for the association between air pollutants (CO and NO_2_) and GN visits in Hefei, China, 2015–2019. **(A)** 3D plot of CO; **(B)** 3D plot of NO_2_. CO, carbon monoxide; NO_2_, nitrogen dioxide; and RR, relative risk. The P_50_ was used as the reference concentration. The P_50_ concentration of CO is 0.8 mg/m^3^ and the P_50_ concentration of NO_2_ is 36 μg/m^3^.

The exposure-response correlation between GN visits and two atmospheric contaminants, CO and NO_2_, reveals a significant positive association at low lag days when concentrations of CO and NO_2_ are high (with reference concentrations of 0.8 mg/m^3^ and 36 μg/m^3^, respectively). However, as the lag time increases, exposure to elevated levels of CO and NO_2_ appeared to diminish the risk of GN visits (Figures 2, 3).

### 3.3. Association between air pollutants and GN visits in a single pollutant model

**Figure 4:**
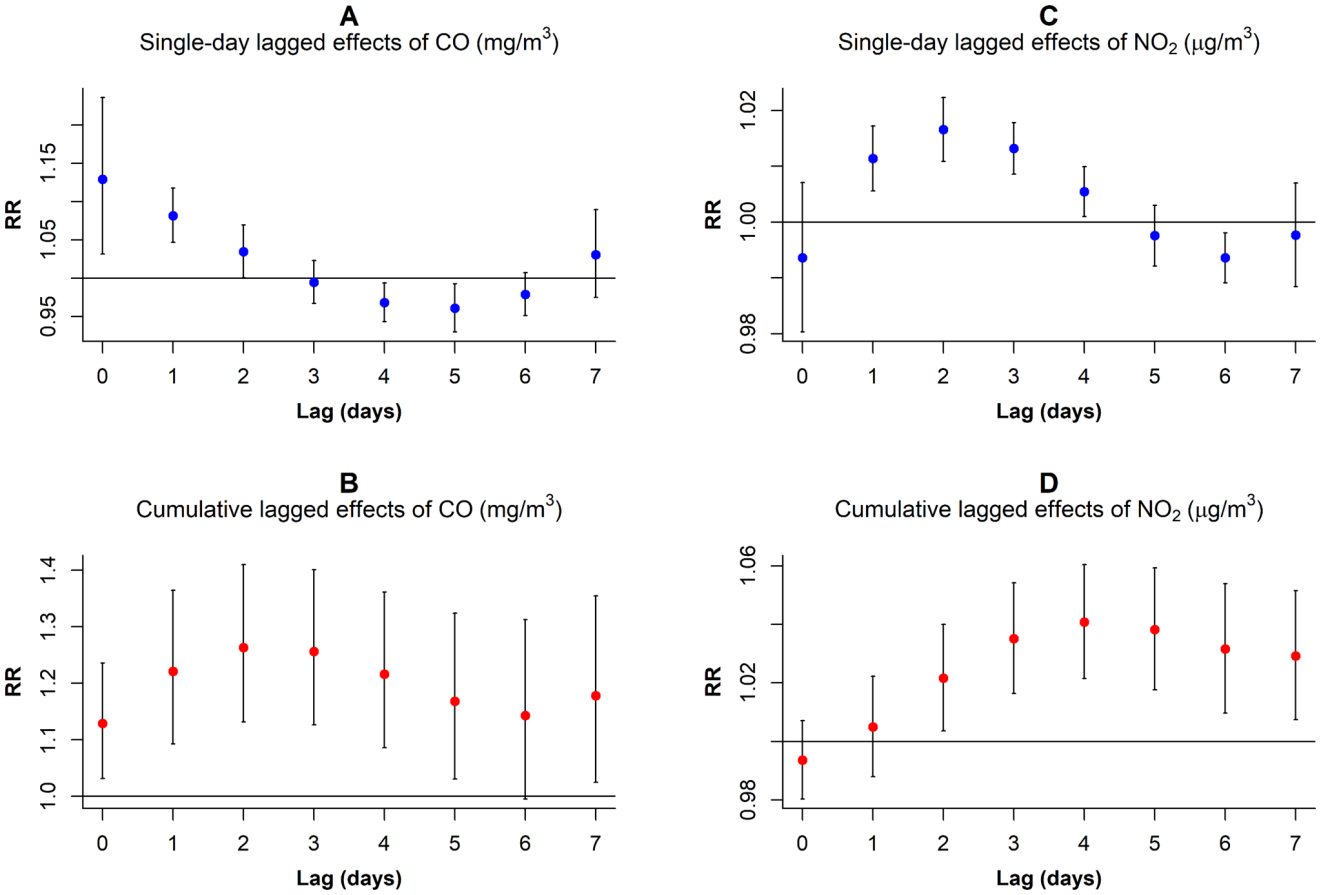
*RR* values and 95% *CI* in the number of daily outpatient visits for GN associated with increases of 1 mg/m^3^ in CO and 10 μg/m^3^ in NO_2_ concentrations at different lag days. **(A)** Single-day lagged effects of CO; **(B)**Cumulative lagged effects of CO; **(C)** Single-day lagged effects of NO_2_; **(D)** Cumulative lagged effects of NO_2_. CO, carbon monoxide; NO_2_, nitrogen dioxide; and RR, relative risk.

Our analysis revealed variations in the RR values corresponding to 1 mg/m^3^ per CO concentration increment and 10 μg/m^3^ per NO_2_ concentration increment across different lag days for GN visitations (Figure 4; Supplementary Table S3). In the single-day lag effect model for CO, the risks for GN visits were positively associated with CO from lag0 (RR: 1.129, 95% CI: 1.031–1.236) to lag2 (RR: 1.034, 95% CI: 1.000–1.070), peaking at lag0. Conversely, the risks decreased at lag4 (RR: 0.968, 95% CI: 0.943–0.993) and lag5 (RR: 0.961, 95% CI: 0.930–0.993). Cumulative lagged effects analysis of CO revealed increased risks of GN visits for all lag days except lag06, and the risk reached its maximum value at lag02 (RR: 1.263, 95% CI: 1.132–1.410).

In contrast to CO, the harm of NO_2_ came more slowly. When considering a single-day lag, increased NO_2_ exposure elevated GN visit risk during the preceding 4 days (lag1–lag4), peaking at lag2 (RR: 1.017, 95% CI: 1.011–1.022). However, the risk decreased at lag6 (RR: 0.994, 95% CI: 0.989–0.998). Furthermore, cumulative lag models showed harmful NO_2_ effects persisting from lag02 to lag07, with the highest risk observed on lag04 (RR: 1.041, 95% CI: 1.021–1.060).

### 3.4. Association between air pollutants and GN visits in a dual-pollutant model

**Figure 5:**
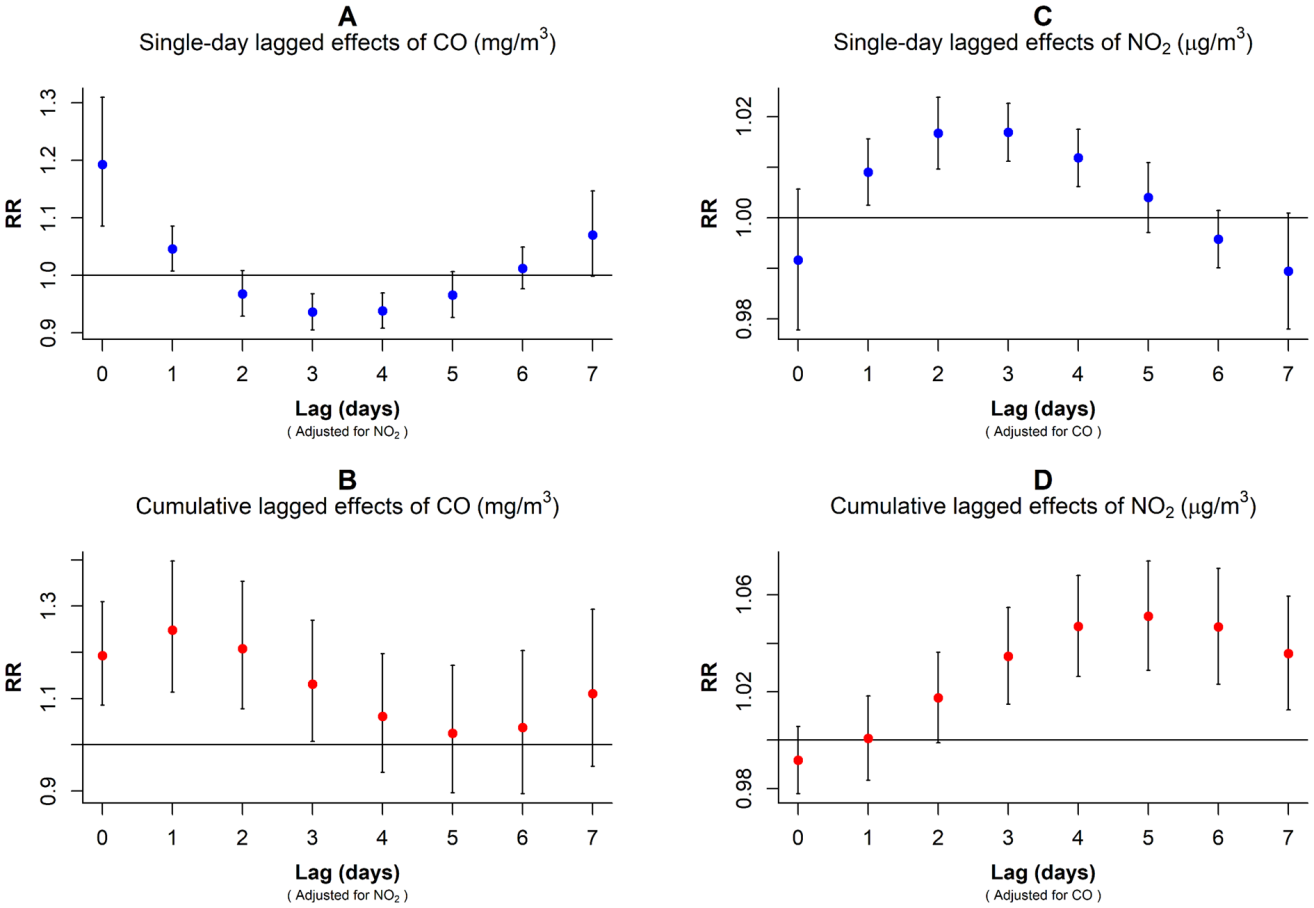
*RR* values and 95% *CI* in the number of daily outpatient visits for GN associated with increases of 1 mg/m^3^ in CO and 10 μg/m^3^ in NO_2_ concentrations in the dual-pollutant model. **(A,B)** Harmful effects of CO exposure after correcting for NO_2_ interference. **(C,D)** Harmful effects of NO_2_ exposure after correcting for CO interference. CO, carbon monoxide; NO_2_, nitrogen dioxide; and RR, relative risk.

In the dual-pollutant model, the significance of NO_2_ and CO persisted when controlling for each other. With NO_2_ adjustment, the single-day lagged effect curve of CO exposure on GN risk exhibited a U-shaped pattern, peaking on exposure day (RR: 1.193, 95% CI: 1.086–1.310) and at the lowest point on the lag3 (RR: 0.936, 95% CI: 0.905–0.968; Figure 5A; Supplementary Table S4). In the cumulative lag model, CO exposure only increased GN visits during the first 3 days (lag01–lag03; Figure 5B; Supplementary Table S4).

After adjusting for CO, the single-day lagged effect trend of NO_2_ followed an inverted U-shape with the prolongation of lag days, peaking at lag3 (RR: 1.017, 95% CI: 1.011–1.023; Figure 5C; Supplementary Table S4). In the cumulative lagged effect model of NO_2_, lag03–lag07 were positively associated with GN visit risk, and the RR value peaked at lag05 (RR: 1.051, 95% CI: 1.029–1.074; Figure 5D; Supplementary Table S4).

### 3.5. Subgroup analysis

**Figure 6:**
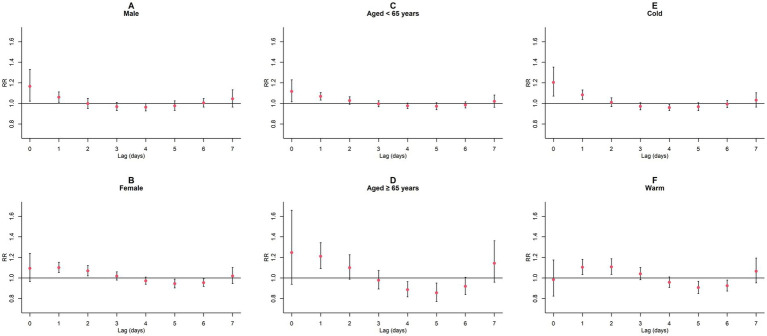
Single-day lagged *RR* values and 95% *CI* of GN visits per 1 mg/m^3^ increase in CO concentration in a model stratified by gender, age, and season. **(A,B)** Single-day lagged effects of CO grouped by gender; **(C,D)** Single-day lagged effects of CO grouped by age; **(E,F)** Single-day lagged effects of CO grouped by season. Harmful effects of CO stratified by sex, age, and season.

**Figure 7:**
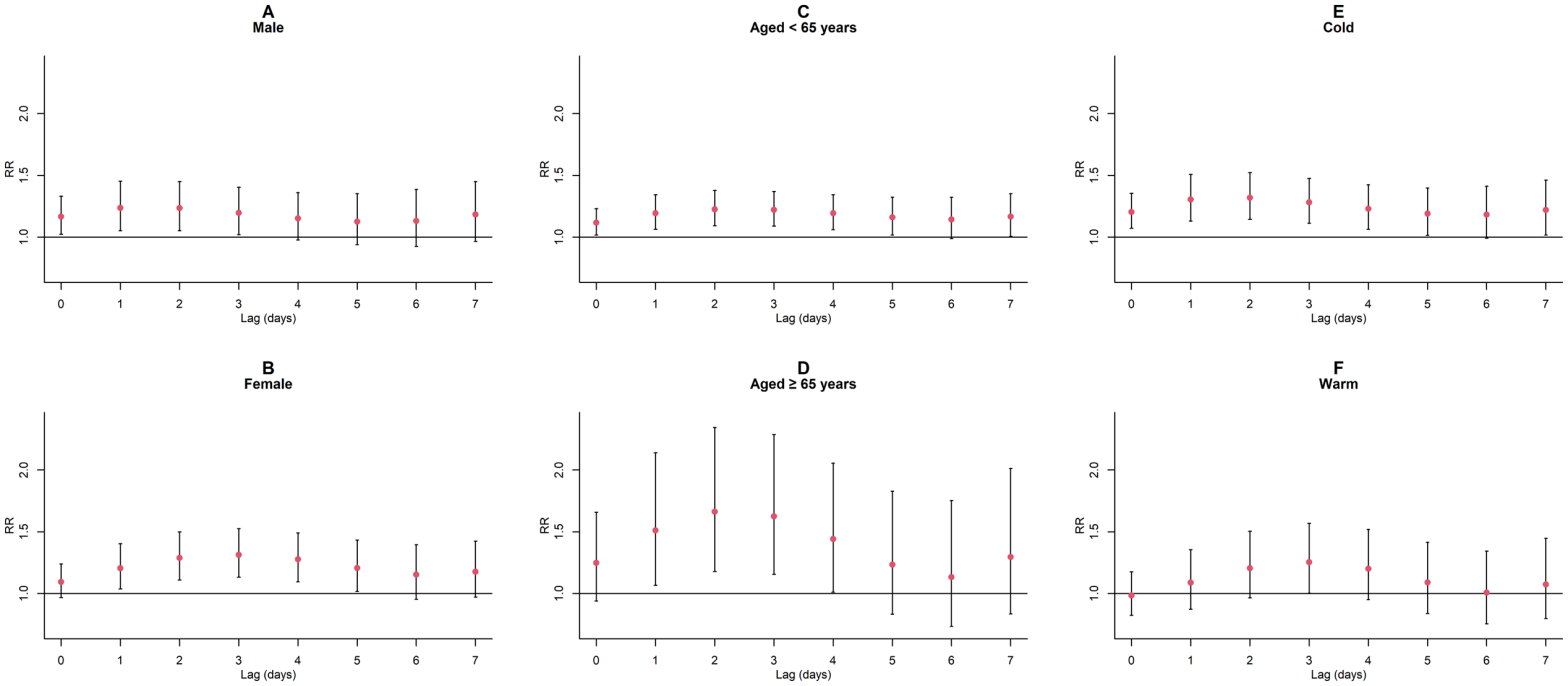
Cumulative lagged *RR* values and 95% *CI* of GN visits per 1 mg/m^3^ increase in CO concentration in a model stratified by gender, age, and season. **(A,B)** Cumulative lagged effects of CO grouped by gender; **(C,D)** Cumulative lagged effects of CO grouped by age; **(E,F)** Cumulative lagged effects of CO grouped by season. Harmful effects of CO stratified by sex, age, and season.

**Figure 8:**
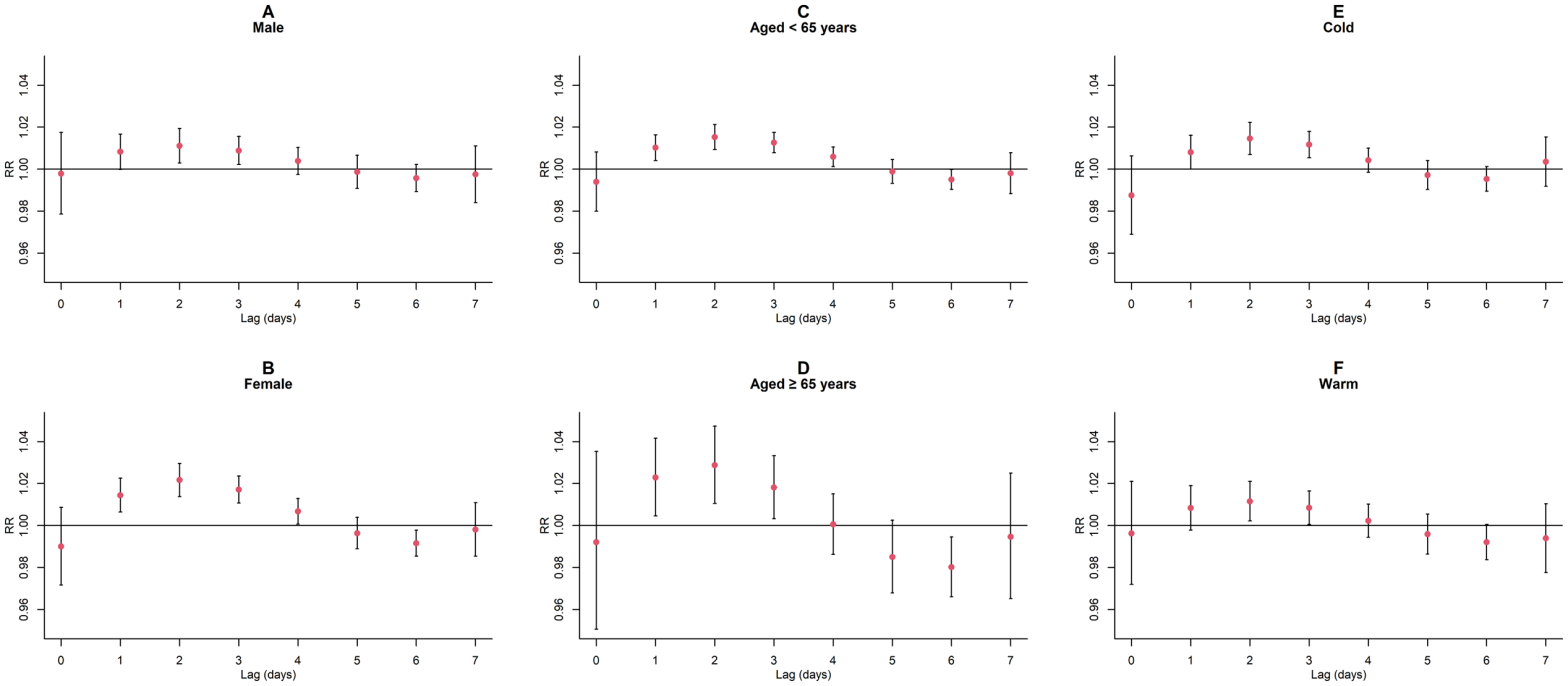
Single-day lagged *RR* values and 95% *CI* of GN visits per 10 μg/m^3^ increase in NO_2_ concentration in a model stratified by gender, age, and season. **(A,B)** Single-day lagged effects of NO_2_ grouped by gender; **(C,D)** Single-day lagged effects of NO_2_ grouped by age; **(E,F)** Single-day lagged effects of NO_2_ grouped by season. Harmful effects of NO_2_ stratified by sex, age, and season.

**Figure 9:**
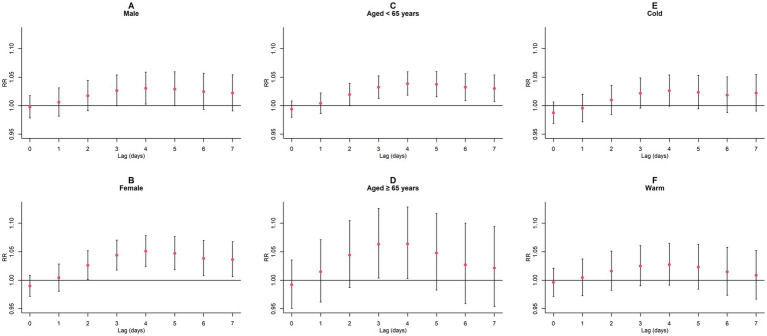
Cumulative lagged *RR* values and 95% *CI* of GN visits per 10 μg/m^3^ increase in NO_2_ concentration in a model stratified by gender, age, and season. **(A,B)** Cumulative lagged effects of NO_2_ grouped by gender; **(C,D)** Cumulative lagged effects of NO_2_ grouped by age; **(E,F)** Cumulative lagged effects of NO_2_ grouped by season. Harmful effects of NO_2_ stratified by sex, age, and season.

Upon conducting stratified analyses based on sex, age, and season, we found that contact with CO and NO_2_ was linked to an increased risk of GN visits across all subgroups (all *p* < 0.05) except for the cumulative lagged effect of the seasonal grouping of NO_2_ (Supplementary Tables S4–S6; Figures 6–9). However, our results from the Wilcoxon signed-rank test showed that only the differences in cumulative lag effect for the age group, season group of CO, and gender group of NO_2_ had statistically significant (Supplementary Table S7). Notably, the older adult and cold-season groups exhibited a heightened susceptibility to the cumulative lagged effect of CO exposure compared to age < 65 years and warm-season groups. The female group was more vulnerable to the harmful effects of cumulative exposure to NO_2_.

### 3.6. Sensitivity analyses

The AIC values of the model when choosing different *dfs* (3–8) for *time*, *air pollutions*, and *meteorological factors* are presented in Supplementary Table S8. In the CO and NO_2_ models, the AIC value is both the smallest (CO: 11250.67; NO_2_:11246.88) when the *dfs* for *time*, *air pollutions*, and *meteorological factors* are 7, 5, and 7, respectively.

Sensitivity analysis results indicated that the consequences of CO and NO_2_ contact on the risk of GN visits remain generally robust after varying the *dfs* of the *time*, *air pollutions*, and *meteorological factors* (Supplementary Figures S4–S9) and regulating the maximum lag periods for CO and NO_2_ (Supplementary Figures S10, S11). After setting the reference values of each pollutant model to 0, our overall exposure effect results remained unchanged (Supplementary Figures S3, S12). Furthermore, the subsequent analyses of single-day and cumulative lag effects for CO and NO_2_ were highly consistent with our previous findings (Figure 4; Supplementary Figure S13; Supplementary Tables S3, S9).

## 4. Discussion

In this investigation, we analyzed nephritis outpatient records from the two prominent grade A tertiary hospitals from 2015 to 2019 to quantitatively assess the correlation between atmospheric contaminants and GN visits. Our findings indicated that contact with CO and NO_2_ were significantly associated with an elevated risk of GN-related hospital visits, suggesting that these pollutants might be a potential risk factor for GN. For each 1 mg/m^3^ elevation in CO level, the GN visit risk escalated by 12.9% in the single-day lag model (lag0, 95% CI: 3.1–23.6%) and by up to 26.3% in the cumulative lag model (lag02, 95% CI: 13.2–41.0%). Meanwhile, every 10 μg/m^3^ elevation in NO_2_ concentration resulted in a 1.7% increase in GN visit risk (lag2, 95% CI: 1.1–2.2%) in the single-day lag model and a 4.1% increase in the cumulative lag model (lag04, 95% CI: 2.1–6.0%).

Previous epidemiological studies examining the correlation between atmospheric contaminants and GN incidence are scarce, focusing more on CKD or ESRD. Our results are supported by a study (24) conducted on a national cohort of US veterans, which observed a positive correlation between increased interquartile ranges (IQR) of CO and NO_2_ concentrations and the decline of glomerular filtration rate, as well as the incidence and progression of CKD and ESRD. However, some studies (25, 26) reported damages of CO exposure on kidney function without identifying an association between kidney disease and NO_2_ exposure. A retrospective cohort research (25) of CKD patients in Seoul, South Korea, confirmed the significant correlation between CO and PM_2.5_ and the long-term mortality risks in CKD patients, but no effects of other pollutants (e.g., NO_2_) on CKD patient mortality were observed. Another study (26) from Korea found that exposure to CO and SO_2_ had the most severe perniciousness on CKD clinic visits. In contrast, studies by Łukasz Kuźma et al. (27) and Szu-Ying Chen et al. (28) identified increased mean levers of NO_2_ and PM_2.5_ as relevant factors for the prevalence and progression of CKD in Bialystok, Poland, and Chinese Taipei, respectively. Interestingly, a national study (29) based on the Chinese CKD survey only demonstrated that long-term exposure to O_3_ elevated the risk of CKD in the general Chinese population. The discrepancies and heterogeneity between these studies may arise from geographical variations, participant sizes, data sources, exposure assessment methods, and distinct air pollution conditions across countries. There are several plausible explanations for the discrepancies between our findings and those of other studies: (1) Substantial differences in air pollution levels between studies could result in the absence of correlation, complicating the comparison of results. For instance, the median annual mean levers of NO_2_ and PM_2.5_ in Bialystok, Poland, were 13.1 and 10.9 μg/m^3^, respectively (27), while in Taipei, China, the annual average concentrations were 24.3 and 23.5 μg/m^3^, respectively (28). Throughout the study period in Hefei City, the levels of NO_2_, CO, PM_10_, PM_2.5_, SO_2_, and O_3_-8h were 40.31 μg/m^3^, 0.84 mg/m^3^, 76.17 μg/m^3^, 52.29 μg/m^3^, 10.55 μg/m^3^, and 86.32 μg/m^3^, respectively. These considerable differences can partially account for the lack of a significant correlation between PM_2.5_ exposure and our study’s increased risk of GN visits. (2) The effects of participants’ social and economic status and risky behaviors may also influence the results. Individuals in countries or regions with higher *per capita* income typically have better education and higher income, potentially providing protective effects against the development of kidney disease (30–32). (3) Variations in disease definitions and diagnostic criteria used across studies may lead to inconsistent results. Although GN ranks as the third and second primary cause of CKD (2) and ESRD (3), the biochemical markers and clinical manifestations used for diagnosis are not identical. Furthermore, the data types and study designs employed in different studies could also affect the results. For example, some studies utilized cross-sectional designs and generalized additive models, while our study adopted time series analysis and DLNM, which might have contributed to differences in the results between studies.

Previous research on air pollution and public health had commonly employed single-pollutant models, neglecting the complex interplay among various pollutants and their collective impact on environmental and human health. In this study, we incorporated both CO and NO_2_ into our model framework. After adjusting for NO_2_ interference, we observed a reduction in the lagged impacts of CO exposure on the risk of GN visits. The RR values diminished more swiftly with increasing lag time but still peaked on the day of exposure (lag0) in the single-day lag model. This phenomenon aligns with previous research (26) examining emergency room visits due to kidney disease concerning ambient air pollution in Korea. In order to examine whether this phenomenon is due to the harvesting effect (22, 23), we reevaluated the risk correlation between CO exposure and GN visits for extended maximum lag days (14 and 21), as depicted in Supplementary Figure S10. The absence of a cosine-like plotting pattern was observed, indicating that it is unlikely that the harvesting effect would have a substantial impact on our estimates. Correspondingly, after controlling for CO interference, the detrimental effects of NO_2_ exposure manifested later, and the decline in RR values decelerated compared to the single-pollutant model. This phenomenon may be attributed to the differential absorption and metabolism rates of various air pollutants in the human body. For instance, a CO inhalation experiment (33) indicated rapid blood CO saturation within 4–5 h, while a NO_2_ inhalation study (34) demonstrated a more gradual response to exposure. Therefore, it is essential to recognize that high CO concentrations may significantly elevate the risk of GN visits and various comorbidities on the day of exposure, while increased attention should be given to the harmful effects of NO_2_ in the days following exposure.

Gender, age, and season are considered influential factors in health assessments and are often standardized as appropriate stratification methods within populations. In this study, we identified compelling results among various subpopulations: females were found to be more susceptible to the effects of NO_2_ than males. This observation may be attributed to the fact that non-smokers are more sensitive to air pollution than smokers (35, 36), and that smoking rates among females are much lower than among males in China (37). Furthermore, regarding the association between age and GN visit risk, CO exhibited more excellent detrimental effects on older than younger individuals. This finding aligns with previous researches (38, 39) examining the link between atmospheric contaminants and disease. The underlying cause may be the higher prevalence of chronic diseases such as diabetes (40), and heart disease (41) among older adults, which are linked to the nosogenesis of kidney diseases (42). Concurrently, CO exposure increases the risk of developing diabetes (43, 44), and heart disease (45, 46). These discoveries emphasize the importance of controlling health indicators such as blood glucose and blood pressure levels to maintain kidney function in older adults. In addition, CO exposure was more strongly associated with GN visits during the cold season. This observation could be explained by the physiological adaptations under colder conditions, where the human body’s blood vessels constriction, intensifying renal ischemia and decreasing renal resistance to both hypoxia and harmful substances (47). Consequently, susceptible populations in highly polluted environments during the cold season, such as females and older individuals, should adopt additional safeguard procedures to alleviate the deleterious influences of atmospheric contaminants on their health.

Various potential biological mechanisms may help elucidate the connection between NO_2_ and CO exposure and the elevated risk of GN visits. CO readily binds to hemoglobin, increasing its affinity for oxygen and subsequently leading to tissue hypoxia (48). Insufficient renal oxygen supply may cause glomerular capillary constriction, impacting filtration function and decreasing glomerular filtration rate (47). Furthermore, tissue hypoxia may result in tubular dysfunction. Animal studies (49) have demonstrated that energy metabolism in renal tubular epithelial cells is impaired under oxygen-deprived conditions, weakening the reabsorption and secretion of filtrate. Additionally, renal hypoxia can stimulate excessive matrix protein production, leading to tissue hardening, functional impairment, and interstitial fibrosis (50). Compared to CO, the biological mechanisms underlying renal injury caused by NO_2_ exposure are more complex. Firstly, NO_2_ may induce renal damage by increasing oxidative stress responses. Toxicological evidence (51) from an animal model suggests that NO_2_ exposure elevates oxidative stress reactions. Moreover, Mirowsky et al. (52) found that genes associated with oxidative stress were highly expressed in primary human bronchial epithelial cells under NO_2_ induction, while substantial evidence (53–55) indicates that oxidative stress has detrimental effects on the kidneys. Secondly, toxicological evidence (56, 57) from animal experiments implies that NO_2_ might straight damage renal function by augmenting the risk of glomerular damage, expansion, hyperfiltration, and heightening susceptibility to infection. Lastly, NO_2_ can activate immune cells, inducing the production of pro-inflammatory cytokines (e.g., TNF-α, IL-1β) (58–60), leading to renal inflammatory responses (61–63).

As an observational investigation, our research presents several limitations that should be considered. Firstly, it is unable to establish causality between atmospheric contaminants and the GN visit risk, and the findings may be subject to residual confounding. Moreover, our study is concentrated solely on Hefei and due to its unique geographical and environmental attributes, the findings might not be universally applicable to other areas. Lastly, we used fixed-site monitoring data to assess air pollution exposure, which does not provide information about individual or indoor exposure levels. Future research should prioritize comprehensive, multi-city analyses and the collection of personal exposure data to assess better potential health risks associated with air pollution exposure.

Notwithstanding its limitations, the present investigation holds considerable merit for multiple reasons. To begin with, it represents the first study that quantifies short-term correlations between CO and NO_2_ exposure and the occurrence of GN clinic visits, employing time-series analysis techniques. This exploratory study contributes to the existing body of research in this domain, providing preliminary insights into the potential implications of air pollutants on GN. Furthermore, this study’s outpatient and meteorological datasets are complete. This comprehensive data allowed for the robust examination of the correlations between atmospheric contaminants and the risk of GN visits and the identification of susceptible subpopulations. Consequently, our findings have the potential to inform the optimization of health policies and regulations aimed at mitigating the impact of GN.

## 5. Conclusion

In summary, the present investigation revealed a significant correlation between contact with CO and NO_2_ and the rising risk of GN visits. Notably, the detrimental impact of CO was discernible on the day of exposure, whereas NO_2_’s adverse consequences emerged in subsequent days. Further stratified analysis unveiled that the cumulative harm of CO was greater in cold seasons and older adult groups. Simultaneously, the female population was more susceptible to the harmful effects of cumulative NO2 exposure. Collectively, the findings of this study offer valuable proof that could bolster public health endeavors to mitigate the impacts of GN via synergistic efforts encompassing efficient environmental regulations and preventive approaches.

## Data availability statement

The data analyzed in this study are subject to the following licenses/restrictions: the dataset is not publicly available in the article and can be requested from the authors upon request. However, only the basic data of deidentified individuals in the article are provided, and proposals for data acquisition can be submitted within 36 months after the publication of the article. Requests to access these datasets should be directed to XF, xinyufang@ahmu.edu.cn.

## Author contributions

HC: conceptualization, data curation, formal analysis, investigation, methodology, validation, visualization, writing—original draft, and writing—review and editing. QD: data curation, writing—original draft, and writing—review and editing. HZ, SW, and XZ: writing—review and editing. DY: data curation, funding acquisition, project administration, resources, supervision, and writing—review and editing. XF: data curation and writing—review and editing. All authors contributed to the article and approved the submitted version.

## Funding

This work was funded by Foundation of Anhui Educational Committee (KJ2020A0223); Inflammation and Immune Mediated Diseases Laboratory of Anhui Province Open Project (IMMDL202110); and The Fund of Anhui Provincial Laboratory of Inflammation and Immune Mediated Diseases (Population Epidemiology Study).

## Conflict of interest

The authors declare that the research was conducted in the absence of any commercial or financial relationships that could be construed as a potential conflict of interest.

## Publisher’s note

All claims expressed in this article are solely those of the authors and do not necessarily represent those of their affiliated organizations, or those of the publisher, the editors and the reviewers. Any product that may be evaluated in this article, or claim that may be made by its manufacturer, is not guaranteed or endorsed by the publisher.

## Footnotes

^1^ http://data.cma.cn/
