# Supplementary material for: Short-term association of CO and NO2 with hospital visits for glomerulonephritis in Hefei, China: a time series study
Source: Front Public Health. 2023 Aug 21;11:1239378. doi: 10.3389/fpubh.2023.1239378 (PMC10475946; doi:10.3389/fpubh.2023.1239378)
Supplement: Supplementary file 1 [file Data_Sheet_1.pdf]

Supplementary Material

Short-term association of CO and NO_2 with hospital visits for glomerulonephritis in Hefei, China: A time series study

Haifeng Chen[1,2†], Qiong Duan[3†], Huahui Zhu[1,2], Shuai Wan[1,2], Xinyi Zhao[1,2], Dongqing Ye[1,2*], Xinyu Fang[1,2*]

[1]Department of Epidemiology and Biostatistics, School of Public Health, Anhui Medical University, Hefei, Anhui, 230032, China

[2]Inflammation and Immune Mediated Diseases Laboratory of Anhui Province, Hefei, Anhui, 230032, China

[3]Department of Health Management Center, the First Affiliated Hospital of Anhui Medical University, Hefei, Anhui, 230022, China

[†] Equal contributions as co-first authors

[*] Corresponding author.

Correspondence to Department of Epidemiology and Biostatistics, School of Public Health, Anhui Medical University, Hefei, China. Prof. Xinyu Fang (xinyufang@ahmu.edu.cn) or Prof. Dongqing Ye (ydq@ahmu.edu.cn)

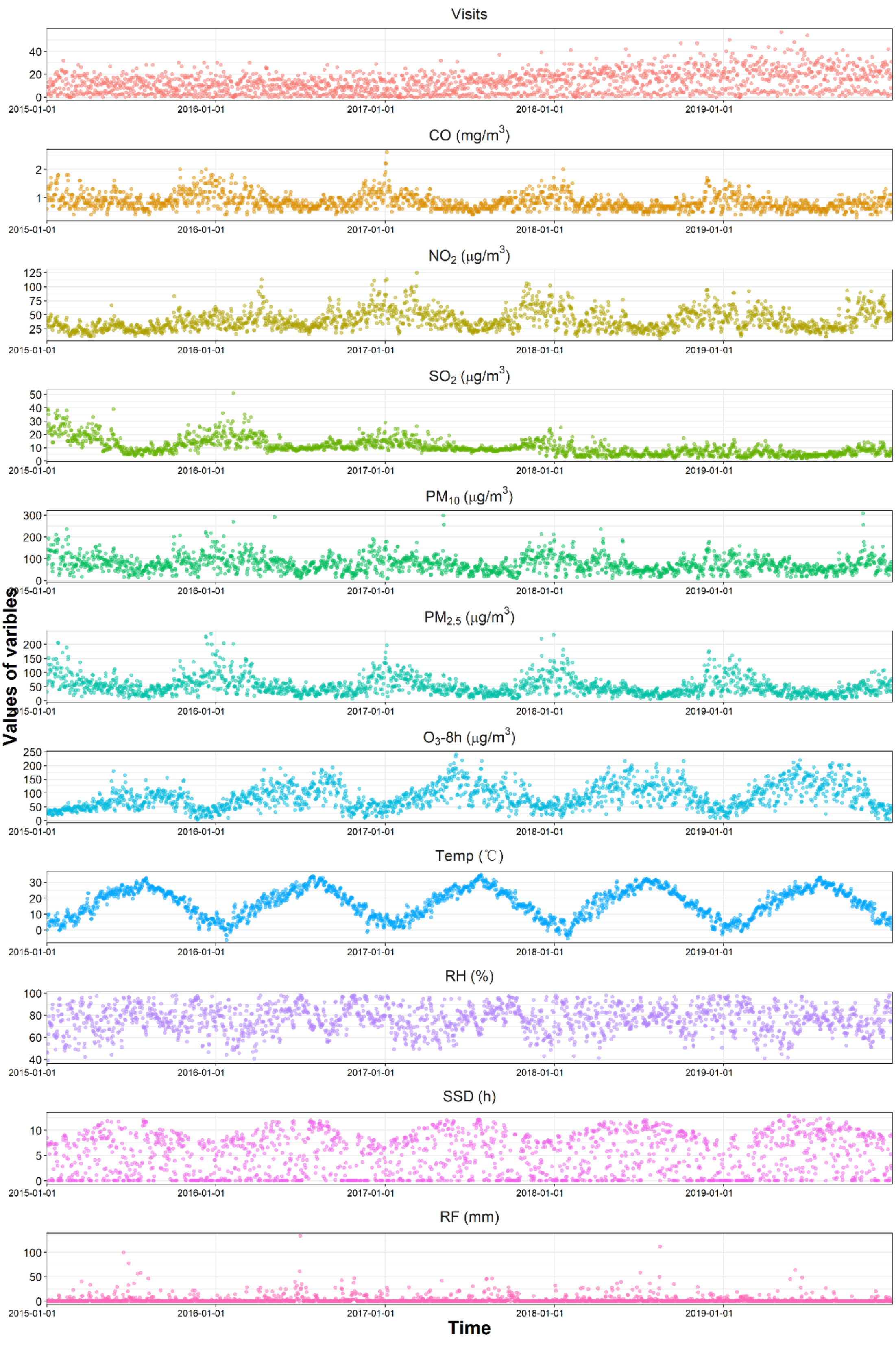

Fig. S1 Time series graph of GN Visits, air pollutants, and meteorological factors in Hefei, China, 2015-2019.

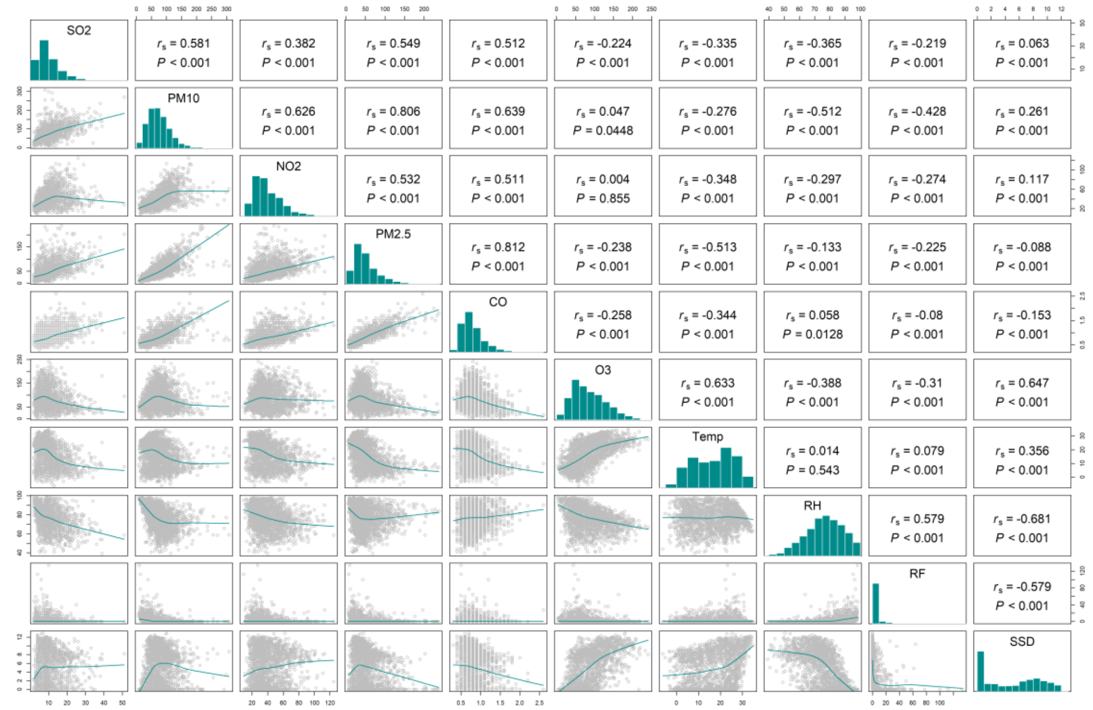

Fig. S2 Spearman correlation analysis between air pollutants and meteorological parameters in Hefei, China, 2015-2019.

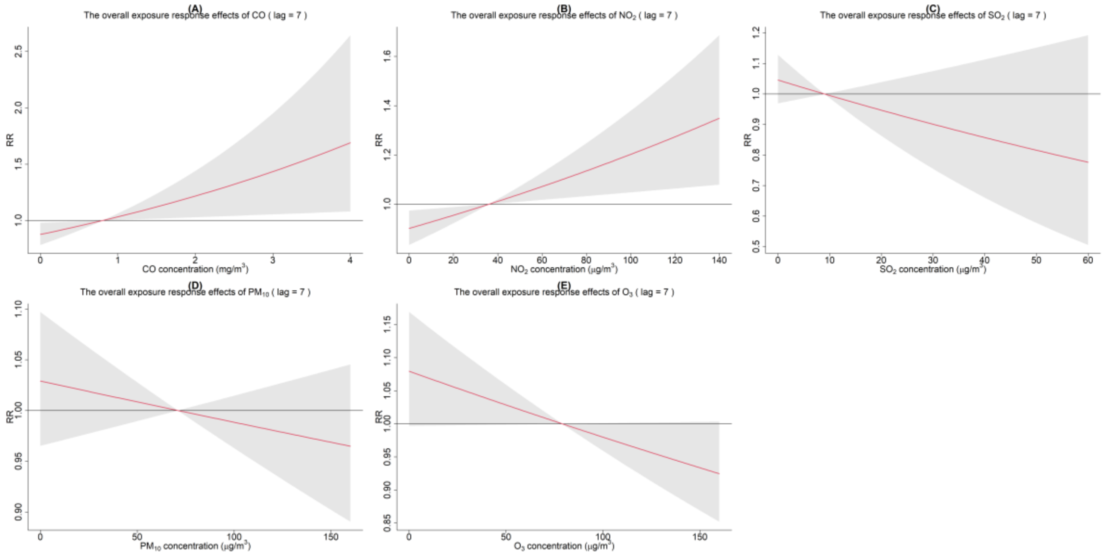

Fig. S3 The overall exposure-response effect curves for associations between air pollutants and GN visits with a lag of 0–7 days in Hefei, China, 2015 to 2019: (A) The overall exposure respbnse effects of CO (lag =7); (B) The overall exposure respbnse effects of NO_2 (lag =7); (C) The overall exposure respbnse effects of SO_2 (lag =7); (D) The overall exposure respbnse effects of PM_{10} (lag =7); (E) The overall exposure respbnse effects of O_3 (lag =7).

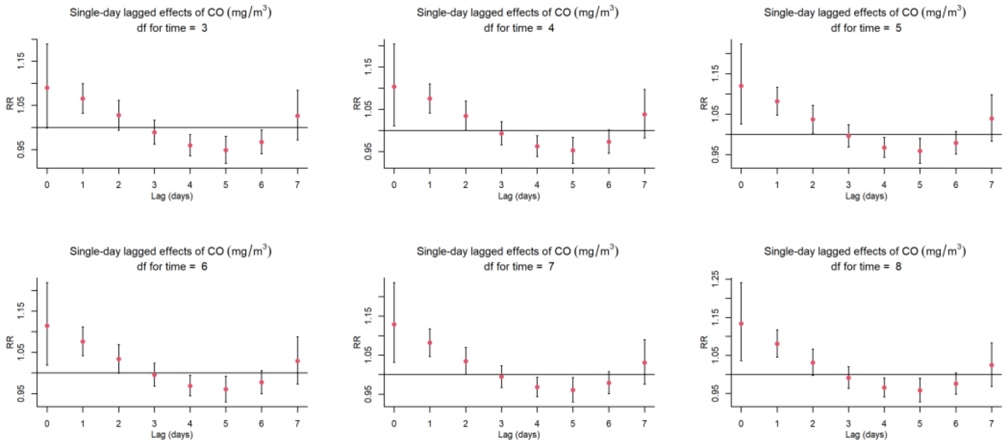

Fig. S4 Single-day lag effects of CO on GN visits under the different *dfs* of *time*.

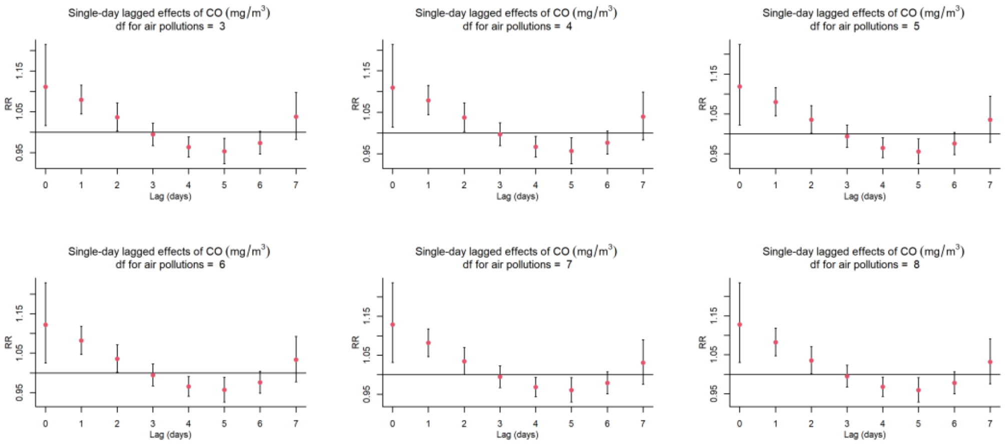

Fig. S5 Single-day lag effects of CO on GN visits under the different *dfs* of *air pollutions*.

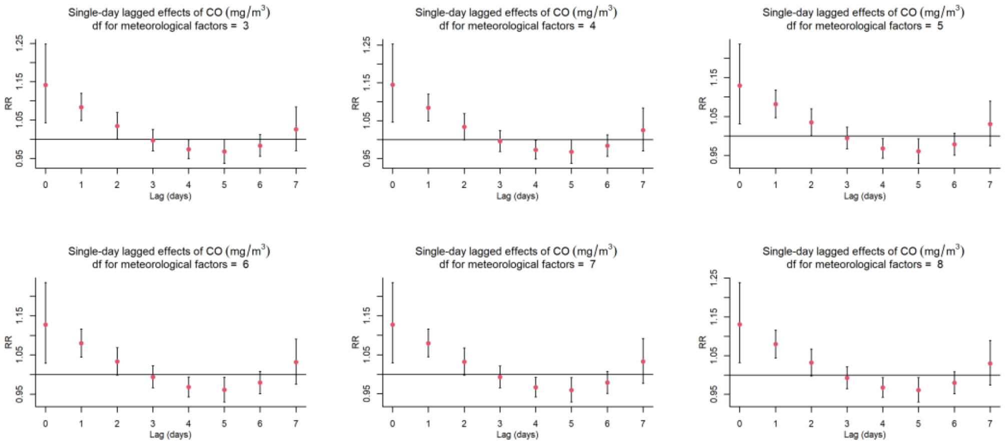

Fig. S6 Single-day lag effects of CO on GN visits under the different *dfs* of *meteorological factors*.

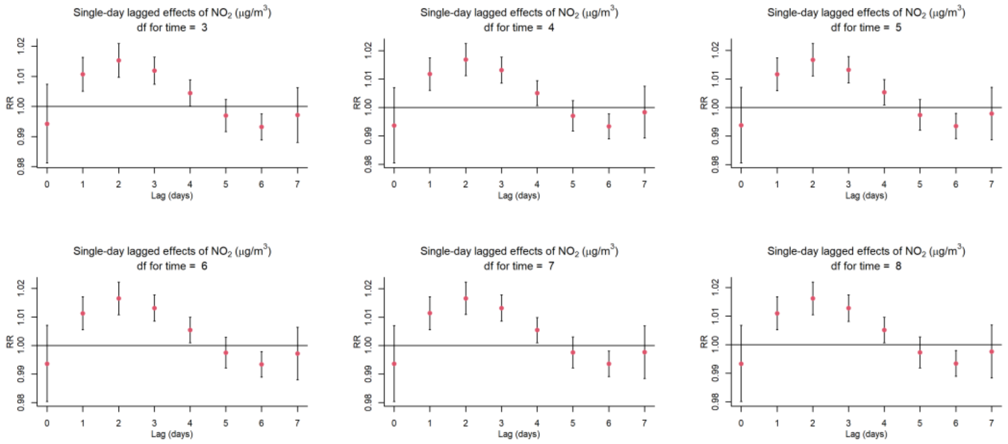

Fig. S7 Single-day lag effects of NO_2 on GN visits under the different *dfs* of *time*.

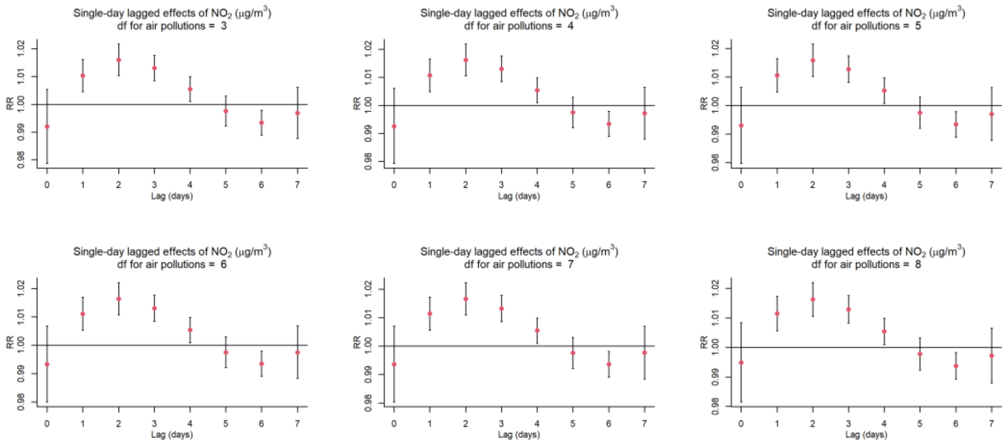

Fig. S8 Single-day lag effects of NO_2 on GN visits under the different *dfs* of *air pollutions*.

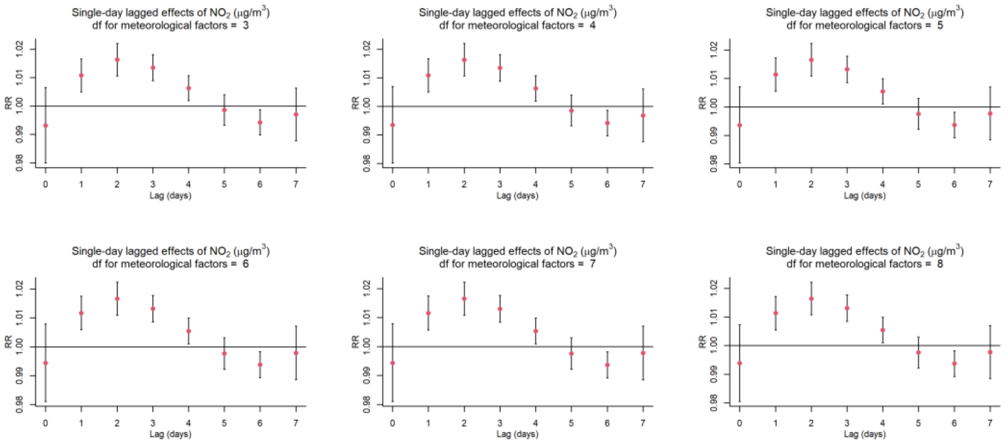

Fig. S9 Single-day lag effects of NO_2 on GN visits under the different *dfs* of *meteorological factors*.

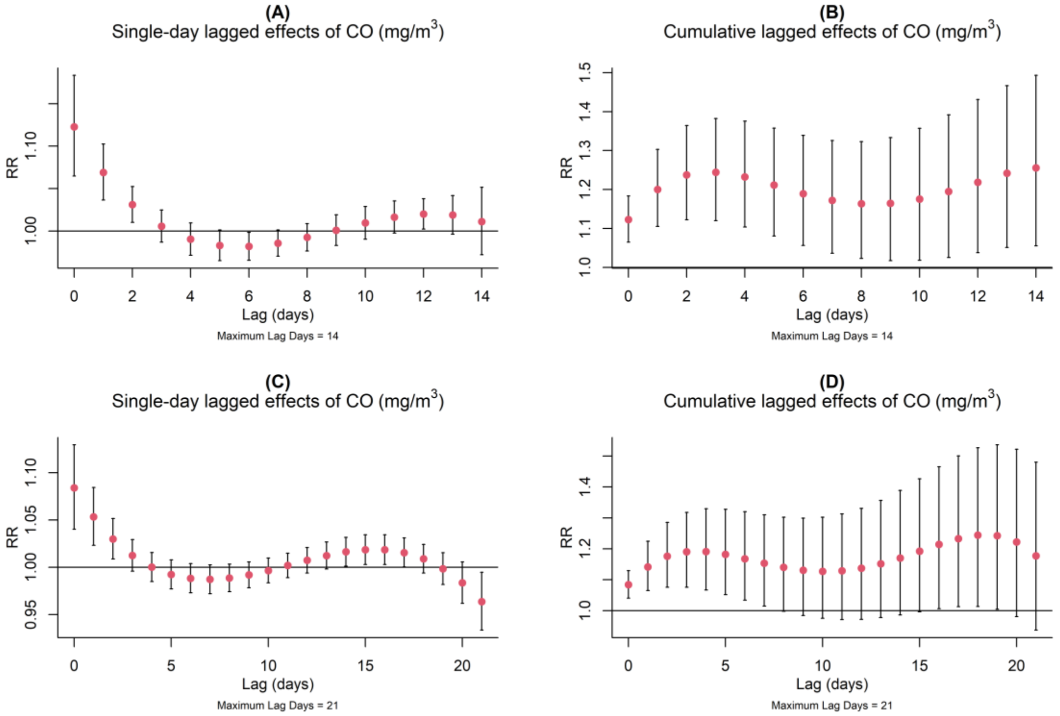

Fig. S10 Single-day lagged effects and cumulative lagged effects of CO and on GN visits under lags 0-14 days and 0-21 days, respectively.

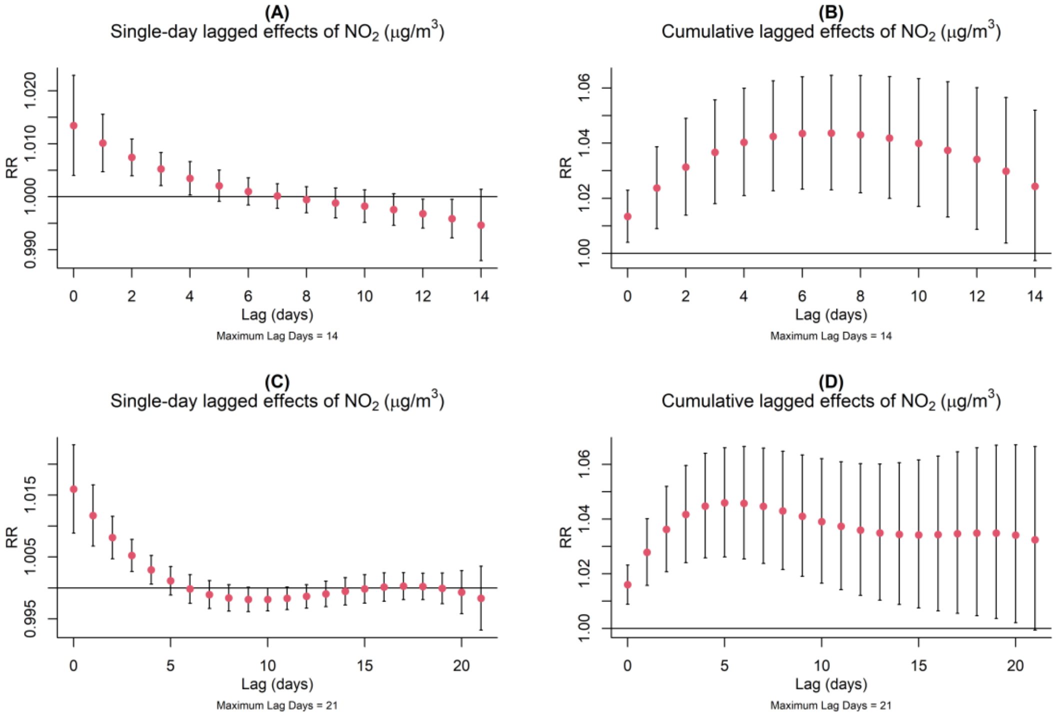

Fig. S11 Single-day lagged effects and cumulative lagged effects of NO_2 and on GN visits under lags 0-14 days and 0-21 days, respectively.

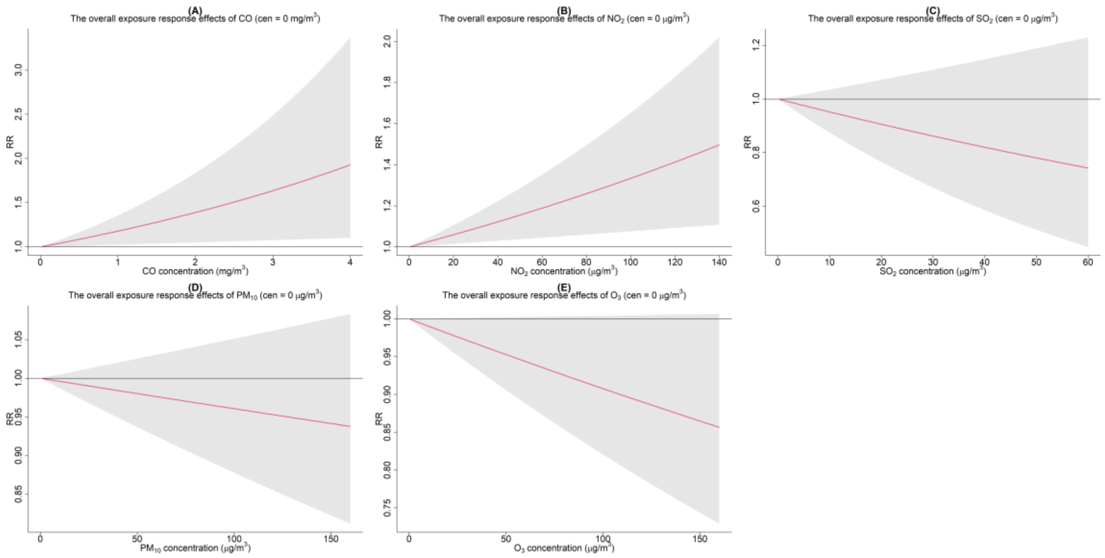

Fig. S12 The overall exposure-response effect curves for associations between air pollutants (reference values = 0) and GN visits with a lag of 0–7 days in Hefei, China, 2015 to 2019: cen: reference values; (A) The overall exposure respbnse effects of CO (lag =7); (B) The overall exposure respbnse effects of NO_2 (lag =7); (C) The overall exposure respbnse effects of SO_2 (lag =7); (D) The overall exposure respbnse effects of PM_{10} (lag =7); (E) The overall exposure respbnse effects of O_3 (lag =7).

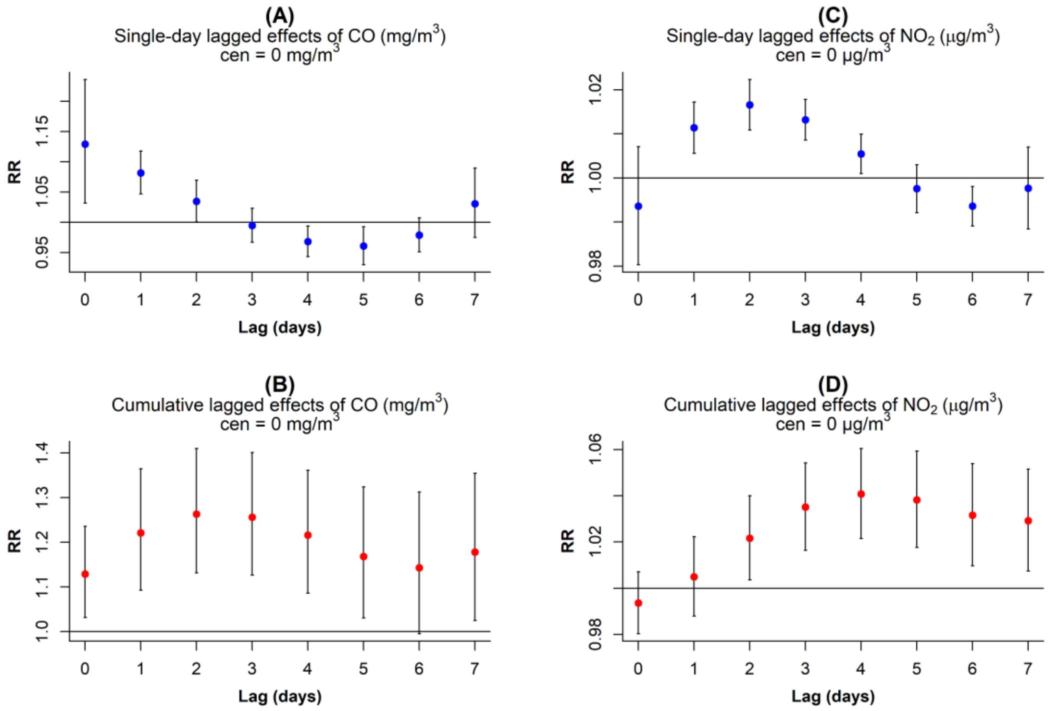

Fig. S13 *RR* values and 95% *CI* in the number of daily outpatient visits for GN associated with increases of 1 mg/m^3 in CO and 10 μg/m^3 in NO_2 concentrations (reference values = 0) at different lag days: cen: reference values.

Table S1 Overdispersion test results of GN visits dataset.

Obs.Var/Theor.Var [a]	Statistic	P
6.94	12,666.58	**< 0.001**

[a] Obs.Var/Theor.Var: The ratio of the observed variance to the theoretical variance. A value >1 indicates that the variance of the observed data is greater than the theoretical variance, suggesting the presence of overdispersion.

Table S2 Normality test results for air pollutants and meteorological factors.

Variable	W	P
SO_2	0.890,426,265	< 0.001
PM_{10}	0.934,859,753	< 0.001
NO_2	0.926,162,244	< 0.001
$PM_{2.5}$	0.867,806,526	< 0.001
CO	0.906,797,167	< 0.001
O_3-8h	0.965,025,470	< 0.001
Temp	0.962,893,396	< 0.001
RH	0.982,958,355	< 0.001
RF	0.397,978,399	< 0.001
SSD	0.892,058,796	< 0.001

Table S3 *RR* value and 95% *CI* in the number of daily outpatient visits for GN associated with increases of 1 mg/m^3 for CO and 10 μg/m^3 for NO_2 concentrations at different lag days.

Lag (d)	CO	NO_2
	RR (95% *CI*)	*RR* (95% *CI*)
Single-day lag		
0	**1.129 (1.031-1.236)** [a]	0.994 (0.980-1.007)
1	**1.082 (1.047-1.118)** [a]	**1.011 (1.006-1.017)** [a]
2	**1.034 (1.000-1.070)** [a]	**1.017 (1.011-1.022)** [a]
3	0.995 (0.967-1.023)	**1.013 (1.009-1.018)** [a]
4	**0.968 (0.943-0.993)** [a]	**1.005 (1.001-1.010)** [a]
5	**0.961 (0.930-0.993)** [a]	0.998 (0.992-1.003)
6	0.979 (0.951-1.007)	**0.994 (0.989-0.998)** [a]
7	1.031 (0.975-1.089)	0.998 (0.988-1.007)
Cumulative lag		
0-0	**1.129 (1.031-1.236)** [a]	0.994 (0.980-1.007)
0-1	**1.221 (1.093-1.364)** [a]	1.005 (0.988-1.022)
0-2	**1.263 (1.132-1.410)** [a]	**1.022 (1.004-1.040)** [a]
0-3	**1.256 (1.127-1.401)** [a]	**1.035 (1.016-1.054)** [a]
0-4	**1.216 (1.086-1.362)** [a]	**1.041 (1.021-1.060)** [a]
0-5	**1.168 (1.031-1.324)** [a]	**1.038 (1.018-1.059)** [a]
0-6	1.143 (0.995-1.313)	**1.032 (1.010-1.054)** [a]
0-7	**1.178 (1.025-1.355)** [a]	**1.029 (1.007-1.052)** [a]

[a] $P < 0.05$

Table S4 *RR* value and 95% *CI* in the number of daily outpatient visits for GN associated with increases of 1 mg/m^3 for CO and 10 μg/m^3 for NO_2 concentrations in the dual-pollutant model.

Lag (d)	CO (Adjusted for NO_2)	NO_2 (Adjusted for CO)
	RR (95% *CI*)	*RR* (95% *CI*)
Single-day lag		
0	**1.193 (1.086-1.310)** [a]	0.992 (0.978-1.006)
1	**1.046 (1.008-1.086)** [a]	**1.009 (1.003-1.016)** [a]
2	0.968 (0.929-1.008)	**1.017 (1.010-1.024)** [a]
3	**0.936 (0.905-0.968)** [a]	**1.017 (1.011-1.023)** [a]
4	**0.938 (0.908-0.969)** [a]	**1.012 (1.006-1.018)** [a]
5	0.966 (0.927-1.006)	1.004 (0.997-1.011)
6	1.012 (0.977-1.049)	0.996 (0.990-1.001)
7	1.070 (0.999-1.147)	0.989 (0.978-1.001)
Cumulative lag		
0-0	**1.193 (1.086-1.310)** [a]	0.992 (0.978-1.006)
0-1	**1.248 (1.114-1.397)** [a]	1.001 (0.983-1.018)
0-2	**1.208 (1.078-1.354)** [a]	1.017 (0.999-1.036)
0-3	**1.131 (1.007-1.270)** [a]	**1.035 (1.015-1.055)** [a]
0-4	1.061 (0.940-1.197)	**1.047 (1.026-1.068)** [a]
0-5	1.025 (0.896-1.172)	**1.051 (1.029-1.074)** [a]
0-6	1.037 (0.894-1.204)	**1.047 (1.023-1.071)** [a]
0-7	1.110 (0.953-1.293)	**1.036 (1.013-1.060)** [a]

[a] $P < 0.05$

Table S5 *RR* value and 95% *CI* of GN visits per 1 mg/m^3 increase for CO concentration in a model stratified by gender, age, and season.

Lag (d)	Male	Female	Age < 65	Age ≥ 65	Cold	Warm
	RR (95% *CI*)	*RR* (95% *CI*)	*RR* (95% *CI*)	*RR* (95% *CI*)	*RR* (95% *CI*)	*RR* (95% *CI*)
Single-day lag						
0	**1.166 (1.022-1.330)** [a]	1.094 (0.966-1.239)	**1.117 (1.015-1.229)** [a]	1.248 (0.939-1.659)	**1.204 (1.071-1.353)** [a]	0.984 (0.823-1.175)
1	**1.060 (1.011-1.113)** [a]	**1.101 (1.053-1.152)** [a]	**1.068 (1.032-1.106)** [a]	**1.211 (1.091-1.343)** [a]	**1.084 (1.039-1.130)** [a]	**1.105 (1.033-1.182)** [a]
2	0.999 (0.951-1.049)	**1.069 (1.021-1.120)** [a]	1.027 (0.991-1.064)	1.100 (0.988-1.225)	1.010 (0.969-1.054)	**1.108 (1.035-1.187)** [a]
3	0.969 (0.930-1.009)	1.019 (0.980-1.060)	0.996 (0.967-1.026)	0.978 (0.893-1.071)	0.972 (0.938-1.007)	1.040 (0.982-1.102)
4	0.963 (0.928-1.000)	0.972 (0.938-1.008)	0.977 (0.951-1.004)	**0.887 (0.816-0.964)** [a]	**0.960 (0.930-0.990)** [a]	0.958 (0.909-1.010)
5	0.977 (0.931-1.025)	**0.945 (0.904-0.989)** [a]	0.972 (0.939-1.007)	**0.856 (0.771-0.950)** [a]	0.968 (0.931-1.007)	**0.907 (0.849-0.968)** [a]
6	1.006 (0.965-1.048)	**0.955 (0.918-0.993)** [a]	0.985 (0.956-1.016)	0.919 (0.838-1.007)	0.993 (0.959-1.029)	**0.925 (0.873-0.980)** [a]
7	1.045 (0.964-1.133)	1.020 (0.945-1.101)	1.020 (0.962-1.081)	1.143 (0.960-1.362)	1.031 (0.964-1.104)	1.066 (0.952-1.193)
Cumulative lag						
0	**1.166 (1.022-1.330)** [a]	1.094 (0.966-1.239)	**1.117 (1.015-1.229)** [a]	1.248 (0.939-1.659)	**1.204 (1.071-1.353)** [a]	0.984 (0.823-1.175)
1	**1.236 (1.051-1.454)** [a]	**1.205 (1.035-1.403)** [a]	**1.193 (1.061-1.341)** [a]	**1.511 (1.067-2.139)** [a]	**1.305 (1.129-1.507)** [a]	1.087 (0.872-1.355)
2	**1.234 (1.051-1.449)** [a]	**1.288 (1.108-1.498)** [a]	**1.226 (1.091-1.376)** [a]	**1.662 (1.178-2.345)** [a]	**1.318 (1.143-1.520)** [a]	1.205 (0.965-1.504)
3	**1.196 (1.020-1.402)** [a]	**1.313 (1.131-1.525)** [a]	**1.221 (1.088-1.369)** [a]	**1.625 (1.154-2.288)** [a]	**1.281 (1.112-1.475)** [a]	**1.253 (1.001-1.569)** [a]
4	1.152 (0.976-1.359)	**1.277 (1.093-1.491)** [a]	**1.192 (1.058-1.344)** [a]	**1.441 (1.010-2.055)** [a]	**1.229 (1.062-1.422)** [a]	1.201 (0.948-1.520)
5	1.125 (0.937-1.351)	**1.207 (1.016-1.433)** [a]	**1.160 (1.016-1.323)** [a]	1.233 (0.832-1.829)	**1.190 (1.013-1.397)** [a]	1.089 (0.838-1.414)
6	1.131 (0.924-1.385)	1.152 (0.953-1.394)	1.143 (0.987-1.322)	1.133 (0.733-1.751)	1.182 (0.989-1.411)	1.007 (0.754-1.344)
7	1.182 (0.965-1.449)	1.176 (0.970-1.424)	1.165 (1.006-1.350)	1.295 (0.834-2.012)	**1.219 (1.016-1.462)** [a]	1.073 (0.796-1.445)

[a] $P < 0.05$

Table S6 Single-day lagged *RR* value and 95% *CI* of GN visits per 10 μg/m^3 increase for NO_2 concentration in a model stratified by gender, age, and season.

Lag (d)	Male	Female	Age < 65	Age ≥ 65	Cold	Warm
	RR (95% *CI*)	*RR* (95% *CI*)	*RR* (95% *CI*)	*RR* (95% *CI*)	*RR* (95% *CI*)	*RR* (95% *CI*)
Single-day lag						
0	0.998 (0.979-1.017)	0.990 (0.972-1.009)	0.994 (0.980-1.008)	0.992 (0.951-1.035)	0.988 (0.969-1.006)	0.996 (0.972-1.021)
1	1.008 (1.000-1.017)	**1.014 (1.006-1.023)** [a]	**1.010 (1.004-1.016)** [a]	**1.023 (1.005-1.042)** [a]	**1.008 (1.000-1.016)** [a]	1.008 (0.998-1.019)
2	**1.011 (1.003-1.019)** [a]	**1.022 (1.014-1.030)** [a]	**1.015 (1.009-1.021)** [a]	**1.029 (1.010-1.047)** [a]	**1.015 (1.007-1.022)** [a]	**1.012 (1.002-1.021)** [a]
3	**1.009 (1.002-1.016)** [a]	**1.017 (1.011-1.024)** [a]	**1.013 (1.008-1.018)** [a]	**1.018 (1.003-1.033)** [a]	**1.012 (1.005-1.018)** [a]	**1.009 (1.001-1.016)** [a]
4	1.004 (0.997-1.010)	**1.007 (1.001-1.013)** [a]	**1.006 (1.001-1.011)** [a]	1.001 (0.986-1.015)	1.004 (0.999-1.010)	1.002 (0.994-1.010)
5	0.999 (0.991-1.007)	0.996 (0.989-1.004)	0.999 (0.993-1.005)	0.985 (0.968-1.003)	0.997 (0.990-1.004)	0.996 (0.986-1.005)
6	0.996 (0.989-1.002)	**0.992 (0.985-0.998)** [a]	**0.995 (0.990-1.000)** [a]	**0.980 (0.966-0.995)** [a]	0.995 (0.990-1.001)	0.992 (0.984-1.001)
7	0.998 (0.984-1.011)	0.998 (0.985-1.011)	0.998 (0.988-1.008)	0.995 (0.965-1.025)	1.004 (0.992-1.015)	0.994 (0.978-1.010)
Cumulative lag						
0	0.998 (0.979-1.017)	0.990 (0.972-1.009)	0.994 (0.980-1.008)	0.992 (0.951-1.035)	0.988 (0.969-1.006)	0.996 (0.972-1.021)
1	1.006 (0.982-1.031)	1.004 (0.981-1.028)	1.004 (0.986-1.022)	1.015 (0.962-1.071)	0.996 (0.972-1.020)	1.005 (0.973-1.037)
2	1.017 (0.991-1.044)	**1.026 (1.001-1.052)** [a]	**1.020 (1.001-1.039)** [a]	1.044 (0.987-1.104)	1.010 (0.985-1.036)	1.016 (0.983-1.051)
3	1.026 (1.000-1.054)	**1.044 (1.018-1.070)** [a]	**1.032 (1.013-1.052)** [a]	**1.063 (1.004-1.126)** [a]	1.022 (0.996-1.049)	1.025 (0.990-1.061)
4	**1.030 (1.003-1.059)** [a]	**1.051 (1.024-1.078)** [a]	**1.039 (1.018-1.059)** [a]	**1.064 (1.003-1.129)** [a]	1.026 (0.999-1.054)	1.027 (0.991-1.064)
5	1.029 (1.000-1.059)	**1.047 (1.018-1.077)** [a]	**1.037 (1.016-1.060)** [a]	1.048 (0.983-1.117)	1.023 (0.994-1.053)	1.023 (0.984-1.063)
6	1.025 (0.993-1.057)	**1.038 (1.008-1.070)** [a]	**1.032 (1.009-1.056)** [a]	1.027 (0.959-1.100)	1.019 (0.988-1.050)	1.015 (0.974-1.058)
7	1.022 (0.991-1.054)	**1.036 (1.006-1.068)** [a]	**1.030 (1.007-1.054)** [a]	1.022 (0.954-1.094)	1.022 (0.991-1.055)	1.009 (0.967-1.052)

[a] $P < 0.05$

Table S7 Results of Wilcoxon signed-rank test for differences between CO and NO_2 subgroups.

Groups	CO		NO_2	
	V [a]	*P*	*V* [a]	*P*
Single-day lag				
gender	19	0.945	24	0.461
age	12	0.461	15	0.742
season	18	1.000	8	0.195
Cumulative lag				
gender	9	0.250	33	**0.039**
age	1	**0.016**	30	0.109
season	36	**0.008**	23	0.547

[a] The statistic V represents the sum of the ranks of the signed differences in the Wilcoxon signed-rank test.

Table S8 The AIC values of the models for CO and NO_2 under different degrees of freedom (*dfs*) of *time*, *air pollutions*, and *meteorological factors*.

dfs	CO			NO_2		
	time	*air pollutions*	*meteorological factors*	*time*	*air pollutions*	*meteorological factors*
3	11275.98	11258.73	11274.56	11274.54	11250.66	11263.64
4	11264.01	11261.14	11262.03	11257.30	11253.56	11262.48
5	11259.26	**11250.67**[a]	11260.64	11253.18	**11246.88**[a]	11261.28
6	11253.99	11254.93	11254.79	11250.18	11249.23	11253.30
7	**11250.67**[a]	11259.23	**11250.67**[a]	**11246.88**[a]	11254.58	**11246.88**[a]
8	11252.30	11261.40	11257.11	11251.21	11256.70	11248.78

[a] The minimum value of AIC.

Table S9 *RR* value and 95% *CI* in the number of daily outpatient visits for GN associated with increases of 1 mg/m^3 for CO and 10 μg/m^3 for NO_2 concentrations (reference values = 0) at different lag days.

Lag (d)	CO	NO_2
	RR (95% *CI*)	*RR* (95% *CI*)
Single-day lag		
0	**1.116 (1.028-1.210)** [a]	0.994 (0.982-1.006)
1	**1.073 (1.042-1.105)** [a]	**1.010 (1.005-1.015)** [a]
2	**1.031 (1.000-1.063)** [a]	**1.015 (1.010-1.020)** [a]
3	0.995 (0.970-1.021)	**1.012 (1.008-1.016)** [a]
4	**0.971 (0.949-0.994)** [a]	**1.005 (1.001-1.009)** [a]
5	**0.964 (0.936-0.993)** [a]	0.998 (0.993-1.003)
6	0.981 (0.956-1.006)	**0.994 (0.990-0.998)** [a]
7	1.028 (0.977-1.080)	0.998 (0.990-1.006)
Cumulative lag		
0-0	**1.116 (1.028-1.210)** [a]	0.994 (0.982-1.006)
0-1	**1.197 (1.083-1.323)** [a]	1.004 (0.989-1.020)
0-2	**1.234 (1.118-1.362)** [a]	**1.019 (1.003-1.036)** [a]
0-3	**1.228 (1.113-1.355)** [a]	**1.032 (1.015-1.049)** [a]
0-4	**1.193 (1.077-1.320)** [a]	**1.037 (1.019-1.054)** [a]
0-5	**1.150 (1.028-1.288)** [a]	**1.034 (1.016-1.053)** [a]
0-6	1.128 (0.996-1.278)	**1.028 (1.009-1.048)** [a]
0-7	**1.159 (1.022-1.314)** [a]	**1.026 (1.007-1.046)** [a]

[a] $P < 0.05$
